# Does Lifelong Exercise Counteract Low-Grade Inflammation Associated with Aging? A Systematic Review and Meta-Analysis

**DOI:** 10.1007/s40279-024-02152-8

**Published:** 2025-01-10

**Authors:** Iñigo M. Pérez-Castillo, Ricardo Rueda, Hakim Bouzamondo, Diego Aparicio-Pascual, Alberto Valiño-Marques, Jose López-Chicharro, Felipe Segura-Ortiz

**Affiliations:** 1 Research and Development, Abbott Nutrition, 68 Camino de Purchil, 18004 Granada, Spain; 2 Research and Development, Abbott Nutrition, Chicago, IL USA; 3 Real Madrid Graduate School, European University of Madrid, Madrid, Spain; 4 Medical Services, Real Madrid, Madrid, Spain

## Abstract

**Background:**

Aging is associated with sustained low-grade inflammation, which has been linked to age-related diseases and mortality. Long-term exercise programs have been shown to be effective to for attenuating this process; however, subsequent detraining might negate some of these benefits. Master athletes, as a model of lifelong consistent exercise practice, have been suggested to present similar inflammatory profiles to untrained young adults. Nonetheless, it is unclear whether maintaining training habits throughout life can completely counteract low-grade inflammation associated with aging.

**Objectives:**

We aimed to systematically evaluate comparisons of baseline inflammatory profiles in Master athletes, untrained middle-aged and older adults, and untrained young individuals to elucidate whether lifelong exercise can counteract low-grade inflammation associated with aging.

**Methods:**

A systematic review was conducted following the Preferred Reporting Items for Systematic Reviews and Meta-Analyses (PRISMA) statement, and a protocol was prospectively registered in PROSPERO (CRD42024521339). Studies reporting baseline systemic levels of proinflammatory and anti-inflammatory markers in Master athletes and untrained controls were eligible for inclusion. A total of six databases (PubMed [MEDLINE], Embase, Cochrane Central Register of Controlled Trials [CENTRAL], Scopus, SPORTDiscus, and Web of Science [WoS]) were searched in September 2024, and studies were independently screened by two reviewers. Risk of bias was assessed using an adapted version of the Joanna Briggs Institute Critical Appraisal tool for cross-sectional trials, and random-effect meta-analyses of standardized mean differences (SMDs) of inflammatory markers were conducted to evaluate comparisons between Master athletes and age-matched untrained middle-aged and older adults as well as Master athletes and young untrained subjects. Subgroup analyses were performed based on exercise intensity and type, and participants’ sex.

**Results:**

A total of 17 studies (*n* = 649 participants) were included both in qualitative and quantitative synthesis. Lifelong exercise appears to attenuate increases in baseline C-reactive protein, and to elevate anti-inflammatory interleukin (IL)-10 levels compared with untrained middle-aged and older adults (C-reactive protein: SMD − 0.71, 95% confidence interval − 0.97, − 0.45, *I*^2^ 0%, *p* = 0.78; IL-10: SMD 1.44, 95% confidence interval 0.55, 2.32, *I*^2^ 87%, *p* < 0.00001). Statistical significance was maintained in C-reactive protein and IL-10 sub-analyses. No difference in tumor necrosis factor-α levels was observed between Master athletes and untrained middle-aged and older adults (SMD 0.40, 95% confidence interval − 0.15, 0.96, *I*^2^ 72%, *p* = 0.0008). A trend towards decreased IL-6 levels in Master athletes was shown in pooled analyses comparing untrained middle-aged and older adults, and rendered statistically significant in sub-analyses. However, comparisons with young untrained adults indicated that Master athletes still present with elevated levels of tumor necrosis factor-α and IL-6, along with decreased IL-10.

**Conclusions:**

Master athletes might exhibit a more anti-inflammatory profile denoted by decreased baseline circulating levels of C-reactive protein and, potentially, IL-6, along with increased IL-10 compared with healthy age-matched untrained peers. However, lifelong exercise might still be insufficient to completely counteract age-related changes in tumor necrosis factor-α, IL-6, and IL-10, as shown in comparisons with untrained young adults.

**Supplementary Information:**

The online version contains supplementary material available at 10.1007/s40279-024-02152-8.

## Key Points

**Table:** 

Master athletes appear to have a lower proinflammatory profile than non-athlete middle-aged and older adults.
Comparisons between Master athletes and untrained younger individuals suggest that lifelong training might not completely counteract low-grade systemic inflammation associated with aging.
Further studies conducting repeated testing, exploring different inflammatory markers, and controlling for potential factors involved, such as dietary practices, are warranted.

## Introduction

While a consensus on the precise definition remains to be established, chronic subclinical inflammation in the absence of overt tissue damage or infection is typically defined as low-grade inflammation [1]. Compared to acute inflammation, systemic low-grade inflammation is characterized by less drastic but sustained increases in circulating levels of inflammatory indicators, namely acute-phase proteins, proinflammatory cytokines and chemokines, soluble adhesion molecules, and leukocytes [2]. A solid body of literature firmly positions changes in inflammation-related markers as a hallmark of metabolic disorders such as diabetes mellitus [3], obesity, [2], and cardiovascular disease [4], among other conditions [5]. Further, not only age-related diseases but also aging per se has been shown to associate with low-grade inflammation, a phenomenon coined “inflammaging”. According to this framework, immune mechanisms endeavoring to neutralize threats throughout life become pervasive with advanced age, a period of life that would not be foreseen by evolution [6]. Although this concept has been revisited in recent years from a more positive perspective [7], the contribution of low-grade inflammation to the progression of age-related diseases and conditions remains unchallenged [5].

Systemic levels of inflammatory markers may not completely encapsulate inflammatory processes and no exhaustive list of inflammatory markers associated with aging is available to date [8]. Nonetheless, cytokines (i.e., interleukin-6 [IL-6] and tumor necrosis factor-α [TNF-α]) and acute phase proteins (i.e., C-reactive protein [CRP]) are commonly analyzed in research to establish the presence of low-grade inflammation, albeit differences across the life span are not always consistent and depend on the biomarker panel explored [9, 10]. Baseline CRP, TNF-α, and IL-6 levels might be of major importance as a substantially higher risk of all-cause mortality with higher levels of these markers has been reported in large cohorts of older individuals [11–13]. As an example, results from a meta-analysis of 14 studies including 83,995 participants indicated that subjects within the highest category of CRP levels measured using high-sensitivity methods (hs-CRP) had a 75% increased risk of all-cause mortality and more than double the risk of cardiovascular mortality compared with those within the lowest category [14]. In the current global landscape marked by aging populations, extending the life span while concurrently preserving good health, often referred to as “health span”, is key, and deeper comprehension of the mechanisms intrinsic to human aging, including low-grade inflammation, coupled with the identification of lifestyle interventions aimed at mitigating these processes, is needed [15].

The mechanisms underlying low-grade inflammation associated with aging remain unclear and several causes have been proposed in the literature including but not limited to the inability of autophagy pathways to remove cellular and molecular debris [16], cell senescence and acquisition of a senescence-associated secretory phenotype [17], and age-related gut dysbiosis [18]. Importantly, unhealthy dietary habits and physical inactivity stand out as the primary modifiable factors believed to exacerbate low-grade inflammation [19, 20]. Regarding the latter, long-term resistance exercise training programs (> 6 weeks, two to three sessions per week) seem to attenuate low-grade inflammation in older adults as indicated by decreased circulating CRP levels, yet changes in different markers might depend on program duration and intensity [21]. In the same vein, a meta-analysis of 11 studies concluded that structured long-term aerobic exercise (> 2 months, two to five sessions per week) appears to attenuate age-related increases in CRP, IL-6, and TNF-α without impacting IL-4 in middle-aged and older adults [22]. Different analyses of the literature have concluded overall anti-inflammatory effects of long-term training in baseline inflammatory profiles of healthy older subjects, which often depend on exercise type, intensity, program duration, and markers assessed [22, 23]. Unfortunately, some evidence suggests that subsequent short-term detraining can substantially negate these changes in inflammatory markers (i.e., IL-6) [24], which raises the question of whether these exercise-induced effects can be maintained throughout life to counteract low-grade inflammation associated with aging.

To answer this question, models of lifelong exercise have been proposed in preclinical research including voluntary running-wheel training and forced treadmill running [25–27]. Following these approaches, one study documented higher anti-inflammatory IL-10, but similar IL-6 and hs-CRP serum levels in lifelong trained compared to sedentary older rats, while another failed to find any association [27]. In another study, Nilsson et al., documented elevations in 17 out of 18 proinflammatory and anti-inflammatory cytokines in older sedentary mice compared with their younger peers, while these increases were largely attenuated in animals subject to voluntary lifelong wheel-running [26]. Regarding humans, the best model for studying exposure to lifelong exercise is Master athletes. These are typically defined as adults aged > 35 years who systematically train for and participate in organized sport competitions [28], and who are characterized by consistently high physical activity patterns throughout life. Importantly, some authors have suggested that Master athletes may have similar resting levels of inflammatory markers to young adults (i.e., ref. [29]); however, this has not been systematically evaluated to date.

In the present work, we aimed to systematically analyze comparisons between baseline inflammatory profiles of Master athletes and untrained middle-aged and older adults to evaluate whether lifelong-trained individuals have a more anti-inflammatory profile than their non-trained age-matched counterparts at rest. Further, comparisons between Master athletes and untrained young adults (healthy controls lacking signs of low-grade inflammation) were systematically evaluated to explore if consistent lifelong exercise habits can counteract low-grade inflammation associated with aging.

## Methods

A systematic review was conducted following the Preferred Reporting Items for Systematic Reviews and Meta-Analyses (PRISMA) 2020 statement [30]. A protocol for the present systematic review was prospectively registered in PROSPERO with the identification number CRD42024521339.

A comprehensive search was conducted by two reviewers in the scientific databases PubMed (MEDLINE), Embase (via ProQuest Dialog^®^), Cochrane Central Register of Controlled Trials (CENTRAL), Scopus, SPORTDiscus, and Web of Science (WoS) in January 2024, and was last updated on 23 September, 2024. Key terms along with Boolean operators and wildcards used in search strings are presented in Table S1 of the Electronic Supplementary Material (ESM). Only English-written peer-reviewed scientific articles published from inception to 23 September, 2024, were included, independently of risk of bias assessment scores. Gray literature (i.e., government reports), and unpublished theses were not specifically screened. All steps involved in record screening process were managed using EndNote X7^®^. References from finally included records were manually searched for potential additional records.

Eligibility criteria for record screening were based on the following population, intervention/phenomenon of interest, comparator, outcomes, and study design (PICOS):

- Population: healthy middle-aged and older athletes of any sex still training at the time of evaluation (mean age > 40 years in reported groups).
- Intervention (phenomenon of interest): at least 10 years of self-reported consistent structured training experience reported either as mean years of training or as study inclusion criterion, independently of the sport discipline performed.
- Comparator: age-matched non-trained healthy adults, or non-trained younger (> 15 years mean difference) healthy controls of any sex.
- Outcomes: baseline circulating levels of proinflammatory or anti-inflammatory cytokines, acute phase proteins (i.e., IL-1β, IL-1ra, IL-4, IL-6, IL-8, IL-10, IL-15, necrosis TNF-α, interferon-γ), CRP, and soluble receptors (sTNF-R1, sTNF-R2, sIL-6R).
- Study design: longitudinal or cross-sectional observational studies, or randomized/non-randomized controlled trials reporting baseline values of inflammatory markers or values obtained prior to placebo/intervention. As data resulting from any potential intervention were not considered in analyses, studies were eligible independently of any randomization process.

Exclusion criteria consisted of studies recruiting only non-athlete subjects or former Master athletes no longer training or participating in competitions, Congress abstracts, studies failing to report critical sociodemographic data (i.e., age) or units for inflammatory markers (i.e., pg/mL), studies reporting only metagenomic or meta-transcriptomic data, and studies exploring only pre-clinical research models, although insights from these records were considered for discussion when available. The screening of retrieved records was performed independently by two reviewers. No calibration exercises were conducted, and agreement exceeded 80% at the full-text level. Any potential disagreement was resolved by a third reviewer.

Data from included studies were extracted independently by two reviewers, and were thoroughly reviewed by a third reviewer to resolve potential discrepancies. A template in Microsoft Excel^®^ was created including the following fields: full citation, study design, inclusion and exclusion criteria, sample size at data analysis, groups of comparison, sociodemographic data (age and sex), years of structured exercise, data on structured exercise (i.e., sports discipline, intensity or type of exercise performed), current training and physical activity habits, potential confounders considered (i.e., dietary intake, smoking habit, and body composition outcomes such as body mass index [BMI] and percentage body fat), biological sample and inflammatory outcomes analyzed, and effect measures (i.e., mean ± standard deviation [SD]).

The risk of bias of included studies was assessed by two reviewers independently, and revised by a third reviewer to resolve any potential disagreement. To this end, an adapted version of the Joanna Briggs Institute Critical Appraisal tool for cross-sectional studies was used [31]. As only the risk of bias at reporting baseline values of inflammatory markers was assessed independently of any potential intervention explored, all included studies were considered to have a cross-sectional design for the purpose of the assessment. The criteria for critical appraisal are presented in Table 1. An overall methodological quality score was assigned based on critical appraisal results as follows: when < 25% of fields were answered “yes”, quality was considered “very low”; between 25 and 50%, quality was considered “low”; between 50 and 75%, quality was considered “moderate”; ≥ 75%, quality was considered “high”.

**Table 1:** Criteria for methodological quality assessment of included studies

JBI field	Were the criteria for inclusion in the sample clearly defined?	Were the study subjects and the setting described in detail?	Was the exposure measured in a valid and reliable way?	Were objective, standard criteria used for measurement of the condition?	Were confounding factors identified?	Were strategies to deal with confounding factors stated?	Were the outcomes measured in a valid and reliable way?	Was appropriate statistical analysis used?
Criteria for high quality (Y)	Inclusion criteria were stated and included age, training history, and health status: Minimum/maximum age or age range was reported for trained subjects and control groups Training/exercise histories were reviewed at recruitment, including sport discipline or type of exercise performed Participants were surveyed, medical history was screened, or health assessments were performed to evaluate health status at recruitment	Study location, recruitment time period, and data on sociodemographic factors (sex, age, and body composition outcomes (i.e., BMI) were reported Physical activity habits of untrained control participants were described	Lifelong exercise was defined based on average number of years of structured exercise experience Data on current training habits were provided (i.e., number of hours of training or distance covered per week, participation in competitions)	Baseline inflammation was evaluated through the assessment of at least two different circulating inflammatory markers	Overweight/obesity, and smoking habits were identified as confounders and excluded from recruitment or considered in analyses Attempts to describe prior exercise/training status and dietary habits (i.e., adherence to specific diets, 24-h recall questionnaires) were done	Analyses were adjusted or subgroup analyses were conducted for at least one potential confounder (i.e., diet, body composition outcomes, exercise type or intensity, sex) when evaluating circulating levels of inflammatory markers	Validated methods were used to quantify inflammatory markers in fasting state	No concern was identified regarding statistical methods approached Sample size calculations were conducted to ensure sufficient statistical power

*BMI* body mass index, *JBI* Joanna Briggs Institute Critical Appraisal tool, *Y* positive answer (yes)

To enable comparisons between groups of study, effect measures of continuous outcomes were retrieved including mean and standard deviation (mean ± SD), mean and standard error (mean ± SE), median and interquartile range [mean (interquartile range)], or log-transformed values of concentration of circulating inflammatory markers at baseline. Prior to analyses, data reported as mean ± SE were transformed to mean ± SD by multiplying the standard error of the mean by the square root of the group sample size. Similarly, mean ± SD values were estimated from mean (interquartile range) data using the mathematical approach proposed by Wan et al. [32]. Whenever log-transformed data were reported, raw mean ± SD values were estimated following Higgins et al. guidelines [33]. Last, whenever data were only available as graphical representations (i.e., bar plots), estimated values were extracted using WebPlotDigitizer^®^ v4.7.

Quantitative data synthesis (meta-analysis) was conducted in Review Manager (RevMan® v5.4) through the calculation of standardized mean differences with 95% confidence intervals [SMD (95% CI)], weighting studies using the inverse variance method. We assumed at the protocol stage that a true effect size was not identical for all included studies, and thus random-effect meta-analyses were planned. All studies were eligible for quantitative synthesis independently of risk of bias assessment scores. Analyses were conducted by pooling SMDs between Master athletes and control groups for each outcome reported in at least three different studies. C-reactive protein and hs-CRP data were pooled a priori in the analyses. Main planned analyses included comparisons between Master athletes and untrained middle-aged and older adults as well as Master athletes and untrained young controls. Additional sub-group analyses were conducted to explore the role of exercise intensity in the levels of inflammatory markers by excluding study arms only consisting of moderately trained athletes as defined by the authors of included studies. In the same vein, studies recruiting only male subjects and studies conducted only in endurance athletes were separately sub-analyzed to explore how the type of exercise and the exclusion of female participants might impact observed results.

Whenever data on different athlete groups (exercise type or intensity) were separately reported for the same outcome, the combined estimate was used in main meta-analyses. In contrast, whenever no combined estimate was reported in the studies, the sample size in the control group was divided by the number of athlete subgroups analyzed to enable pair-wise comparisons. Whenever overlapping of the populations of study was suspected in different records, authors were contacted to clarify the data. Accordingly, only the study with the highest reported number of participants for each outcome was included in the analyses to prevent potential double-counting. Forest plots were created to graphically represent data synthesis results. Effect sizes (pooled SMDs) were considered small (± 0.2), moderate (± 0.5), or large (± 0.8) based on Cohen’s recommendations [34]. The heterogeneity of included studies was assessed using I^2^ statistics. Values > 30%, > 50%, and > 75% were considered as moderate, substantial, and considerable heterogeneity, respectively [35]. As no single pooled analysis included ten or more different studies, publication bias was not explored because of a lack of power to distinguish real funnel plot asymmetry [35].

Last, the quality of the evidence of performed pooled analyses was not assessed as most available methodologies (i.e., Grading of Recommendations Assessment Development, and Evaluation [GRADE] [36]) rate evidence arising from non-randomized trials or observational studies as low or very low quality, which might not accurately represent the evidence herein reviewed.

## Results

**Fig. 1:**
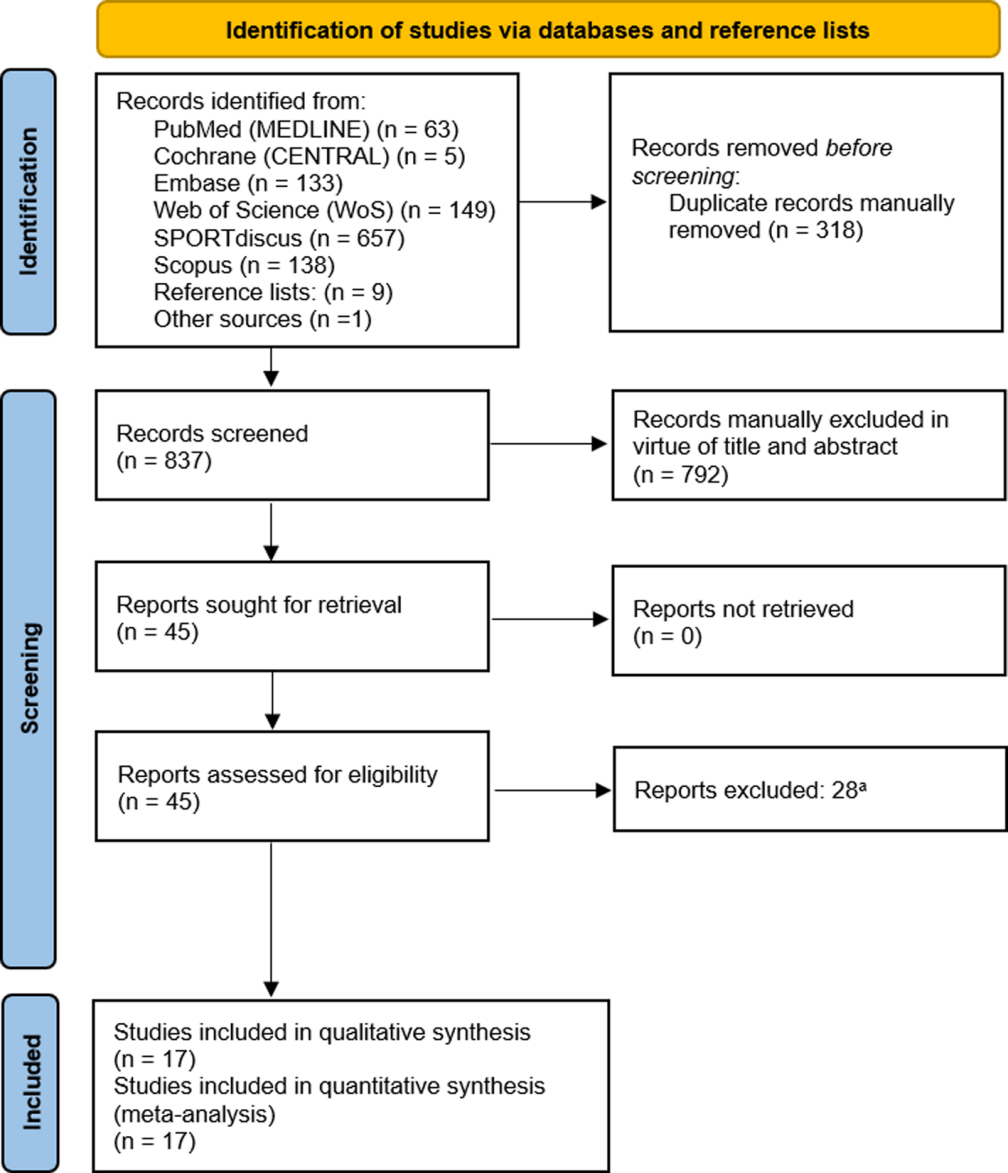
Preferred Reporting Items for Systematic Reviews and Meta-Analyses (PRISMA) flow diagram of record identification, screening, and selection processes. ^a^ Reports excluded and reasons for exclusion are presented in the Electronic Supplementary Material

The record identification, screening, and selection process is summarized in Fig. 1. A total of 1145 records were identified through the searches conducted in six databases. Nine additional records were identified through checking reference lists from included articles, albeit none of them was finally considered eligible for inclusion. One additional record was identified from secondary sources (Google Scholar). Duplicated records were manually removed yielding 837 records available for screening. After removing 792 records by virtue of title and abstract, 45 reports were sought for retrieval, and all of them were retrieved for full-text screening. Last, 28 reports were excluded from this review (Tables S2–S3 of the ESM) leaving a total of 17 studies for qualitive synthesis. Effect measures of inflammatory markers from all 17 included studies were available for meta-analyses.

### Characteristics of the Studies

The characteristics of included studies are presented in Table 2. A total of three reports documented the same study protocol identification number [37–39], and overlapping was suspected for two further records [40, 41]. Therefore, authors were contacted, and the populations of the studies were confirmed to be identical. Accordingly, these records were considered as single studies in terms of data extraction, risk of bias assessment, and quantitative synthesis.

**Table 2:** Characteristics of included studies

Reference	Sample size at baseline analysis and age of participants	Characteristics of participants	Analyzed blood inflammatory markers	Reported results
Aguiar et al., 2020 [37–39]	*n* = 30 MA (18 endurance runners, 12 sprint/power athletes); 51.56 ± 8.61 y *n* = 12 UO; 45.54 ± 9.86 y *n* = 17 UY; 22.70 ± 3.92 y* (mean ± SD)	MA: male; 24.8 ± 9.41 y of training experience; 6.67 ± 3.39 h/wk of training; 6.67 ± 3.78 competitions/y; 21.76 ± 6.12 BMI; 12.77 ± 4.28 percentage body fat assessed using the skinfold technique UO: male; recreationally active; 31.20 ± 8.43 BMI*; 25.72 ± 4.22 percentage body fat* assessed using the skinfold technique UY: male; recreationally active; 23.55 ± 2.20 BMI; 11.47 ± 3.79 percentage body fat assessed using the skinfold technique (mean ± SD)	Fasting baseline circulating IL-6, IL-10, IL-15, IL-17, IL-18, sIL-6R, sTNF-R1, and TNF-α levels quantified using ELISA. No previous exercise in the last > 24 h	MA (combined) vs UO: ↑ IL-10; ↓ IL-6, sIL-6R, and sTNF-R1; = TNF-α MA (combined) vs UY: ↑ IL-6, TNF-α, sIL-6R, and sTNF-R1; ↓ IL-10 MA (endurance) vs UO: ↑ IL-10, IL-15; ↓ IL-6, sIL-6R, and sTNF-R1; = IL-17, IL-18 and TNF-α MA (endurance) vs UY: ↑ IL-6, IL-18, TNF-α, sIL-6R, and sTNF-R1; ↓ IL-10 and IL-17; = IL-15 MA (sprint) vs UO: ↑ IL-10; ↓ IL-6, IL-17, IL-18, sIL-6R, and sTNF-R1; = IL-15 and TNF-α MA (sprint) vs UY: ↑ IL-18, TNF-α, sIL-6R, and sTNF-R1; ↓ IL-15 and IL-17; = IL-6, and IL-10
Barbosa et al., 2021 [49]	*n* = 70 MA (power and endurance athletes) (*subgroups not reported*); 51.3 ± 8 y *n* = 24 UO; 47.2 ± 8 y *n* = 32 UY; 23.7 ± 3.9 y* (mean ± SD)	MA: male; 24.95 ± 9.92 y of training experience; 9.26 ± 3.69 h/wk of training; 8.33 ± 5.45 competitions/y; 23.5 ± 3.7 BMI; 13.4 ± 4.4 percentage body fat assessed using the skinfold technique UO: male; 27.2 ± 2.9 BMI*; 22.1 ± 4.2 percentage body fat* assessed using the skinfold technique UY: male; 24.3 ± 2.7 BMI*; 14.6 ± 4.9 percentage body fat* assessed using the skinfold technique (*physical activity of control groups not reported*) (mean ± SD)	Fasting baseline circulating IL-6, IL-10, and TNF-α levels quantified using ELISA (*previous exercise not specified)*	MA vs UO: ↑ IL-10, and TNF-α; ↓ IL-6 MA vs UY: ↑ IL-6, and TNF-α; ↓ IL-10
Girginer et al., 2019 [43]	*n* = 43 MA (22 high-intensity endurance athletes; 57.28 ± 6.38 y, and 21 moderate-intensity endurance athletes; 62.38 ± 2.12 y) *n* = 17 UO; 52.23 ± 6 y* (mean ± SD)	MA (intense): male; > 10 y of training experience; > 10 h/wk of training; competing regularly; 24.30 ± 1.14 BMI; 9.27 ± 3.82 percentage body fat assessed using bioimpedance MA (moderate): male; > 10 y of training experience; 3 h/wk of training; not competing; 27.20 ± 2.78 BMI*; 19.55 ± 6.01 percentage body fat* assessed using bioimpedance UO: male; sedentary; 26.33 ± 1.1 BMI*; 19.64 ± 2.6 percentage body fat* assessed using bioimpedance (mean ± SD)	Baseline circulating hs-CRP, and TNF-α, levels quantified using ELISA (*previous fasting or exercise not specified*)	MA (intense) vs UO: = hs-CRP, and TNF-α^a^ MA (moderate) vs UO: = hs-CRP, and TNF-α^a^
Gutierrez et al., 2021 [50]	*n* = 25 MA (endurance athletes); 51.48 ± 9.49 y *n* = 23 UO; 46.0 ± 9.37 y (mean ± SD)	MA: male; 21.71 ± 10.19 y of training experience; ≈12 h/wk of training; still competing; 23.19 ± 2.09 BMI; 14.15 ± 3.82 percentage body fat assessed using the skinfold technique UO: male; sedentary; 28.48 ± 4.47 BMI*; 23.42 ± 4.95 percentage body fat* assessed using the skinfold technique (mean ± SD)	Fasting baseline circulating IL-10 levels quantified using ELISA. No previous exercise in the last 24 h	MA vs UO: ↑ IL-10
Hayes et al., 2021 [51]	*n* = 17 MA (endurance athletes and water polo players); 60 ± 5 y *n* = 22 UO; 62 ± 2 y (mean ± SD)	MA: male; > 30 y of training experience; still competing; ≈26.1 BMI UO: male; sedentary; ≈29.7 BMI (*weight differences not analyzed*) (*h/wk of training and body fat values not reported*) (mean ± SD)	Fasting baseline circulating IL-6, and hs-CRP levels quantified using ECLIA. No previous exercise in the last 48–72 h	MA vs UO: ↓ IL-6, and hs-CRP
Lavin et al., 2020 [45]	*n* = 21 MA (primarily endurance athletes; 74 ± 1 y) [14 high intensity; 74 ± 1 y and 7 moderate intensity; 75 ± 2 y]. *n* = 10 UO; 75 ± 1 y (mean ± SE)	MA (combined): male; 53 ± 1 y of training experience; 7.3 ± 0.5 h/wk of training; 14 of 21 competing regularly; 24 ± 1 BMI; 24 ± 1 percentage body fat assessed using DXA MA (intense): male; 53 ± 1 y of training experience; 7.6 ± 0.7 h/wk of training; competing regularly; 24 ± 1 BMI; 22 ± 1 percentage body fat assessed using DXA MA (moderate): male; 53 ± 3 y of training experience; 6.6 ± 0.9 h/wk of training; (*participation in competitions not reported*); 25 ± 1 BMI; 27 ± 1 percentage body fat assessed using DXA UO: male, 5,813 ± 488 steps per day*; 28 ± 1 BMI*; 32 ± 1 percentage body fat* assessed using DXA (mean ± SE)	Fasting baseline circulating CRP levels quantified using LIA, and IL-6 and TNF-α quantified using ELISA. No previous exercise in the last 72 h	MA (combined) vs UO: **↓** IL-6; = CRP and TNF-α MA (intense) vs UO: = CRP, IL-6 and TNF-α MA (moderate) vs UO: = CRP, IL-6 and TNF-α
Lavin et al., 2020 [46]	*n* = 7 MA (primarily endurance athletes); 72 ± 2 y *n* = 10 UO; 75 ± 1 y (mean ± SE)	MA: female; 48 ± 2 y of training experience; 6.6 ± 0.6 h/wk of training; some of them competing regularly; 23 ± 1 BMI; 30 ± 2 percentage body fat assessed using DXA UO: female; 6800 ± 823 steps per day; 27 ± 1 BMI*; 41 ± 2 percentage body fat* assessed using DXA (mean ± SE)	Fasting baseline circulating CRP levels quantified using LIA, and IL-6 and TNF-α quantified using ELISA. No previous exercise in the last 72 h	MA vs UO: = CRP, IL-6, TNF-α
McKendry et al., 2019 [47]	*n* = 14 MA (endurance athletes); 67.1 ± 6.4 y *n* = 12 UO; 69.8 ± 4.1 y *n* = 15 UY; 20.0 ± 2.7 y* (mean ± SD)	MA: males; 36.5 ± 8.1 y of training experience; 7.6 ± 4.7 h/wk of training; 23.0 ± 2.0 BMI; 19.2 ± 4.1 percentage body fat assessed using DXA UO: males; physically active; 24.5 ± 3.8 BMI; 26.8 ± 5.4 percentage body fat* assessed using DXA UY: male; physically active; 24.6 ± 3.6 BMI; 22.0 ± 5.5 percentage body fat assessed using DXA (mean ± SD)	Fasting baseline circulating CRP levels quantified using ELISA. No previous exercise in the last 48 h	MA vs UO: = CRP MA vs UY: = CRP
Mathur et al., 2013 [53]	*n* = 15 MA (endurance athletes); 54 ± 4 y *n* = 17 UO; 55 ± 5 y (mean ± SD)	MA: 10 female, and 5 male; 14 ± 11 y of training experience; 22 ± 5 BMI UO: 8 female, 9 male; sedentary; 26 ± 3 BMI* (mean ± SD) (*h/wk of training and participation in competitions not reported*)	Baseline circulating CRP levels (a*nalytical technique, previous fasting or exercise not specified*)	MA vs UO: ↓ CRP
Mikkelsen et al., 2013 [52]	*n* = 15 MA (endurance athletes); 64 ± 4 y *n* = 12 UO; 66 ± 4 y *n* = 12 UY; 24 ± 3 y* (mean ± SD)	MA: male; 28 ± 2 (mean ± SE) y of training experience; 23 ± 2 BMI (mean ± SD) UO: male; IPAQ score of 1,277 ± 575* (mean ± SE); 25 ± 2 BMI* (mean ± SD) UY: male, IPAQ score of 919 ± 199 (mean ± SE); 22 ± 2 BMI (mean ± SD) (*h/wk of training and participation in competitions not reported*)	Fasting baseline circulating CRP, IL-6, TNF-α, sTNF-R1, and sTNF-R2 levels quantified using ELISA. No previous exercise in the last 24 h	MA vs UO: ↑ TNF-α; = CRP, IL-6, sTNF-R1, and sTNF-R2 MA vs UY: = CRP, IL-6, TNF-α, sTNF-R1, and sTNF-R2
Minuzzi et al., 2019 [40, 41]	*n* = 20 MA (judo, swimming, and athletics); 53.1 ± 8.8 y *n* = 10 UO; 54.2 ± 5.9 y *n* = 9 UY; 31.8 ± 3 y* (mean ± SD)	MA: 3 female, and 16 male (*one not reported*); 24.6 ± 1.83 y of training experience; 5.45 ± 0.42 h/wk of training; competing regularly; 25.1 ± 4.6 BMI UO: 4 female and 6 male; 24.3 ± 3.2 BMI UY: *(sex not reported)*; 21.8 ± 2 BMI *(physical activity of control groups not reported)* (mean ± SD)	Baseline circulating CRP quantified using LIA, and IL-1β, IL-1ra, IL-4, IL-6, IL-8, IL-10, IL-15, IL-17, and TNF-α levels quantified using ELISA. No previous exercise in the last 72 h (*previous fasting not specified*)	MA vs UO: ↑ IL-1β, IL-1ra, IL-4, IL-8, and IL-10; = CRP, IL-6, IL-15, and TNF-α MA vs UY: ↑ IL-1β, IL-1ra, IL-4, and IL-8; ↓ IL-10 and IL-17; = CRP, IL-6, IL-15, and TNF-α
Nickels et al., 2022 [42]	*n* = 23 MA (12 endurance athletes; 45.5 ± 4.9 y, and 11 sprinters; 50 ± 9.1 y) n = 12 UO; 49.2 ± 7 y *n* = 11 UY; 24.1 ± 1.6 y* (mean ± SD)	MA (endurance): 5 female, and 7 male; 19.3 ± 13.3 y of training experience; 6.5 ± 2.3 h/wk of training; still competing; 21.9 ± 1.8 BMI; 15.4 ± 5 percentage body fat assessed using bioimpedance MA (sprint): 6 female, 5 male; 30.9 ± 16.8 y of training experience; 6.9 ± 2.1 h/wk of training; still competing; 24.4 ± 2.7 BMI; 20.3 ± 4.7 percentage body fat assessed using bioimpedance UO: 7 female, 5 male; sedentary; 29.3 ± 2.8 BMI*; 34.6 ± 7.2 percentage body fat* assessed using bioimpedance UY: 5 female, 6 male; sedentary; 22.5 ± 3.4 BMI; 19.4 ± 8 percentage body fat assessed using bioimpedance (mean ± SD)	Baseline circulating CRP, IL-8, and TNF-α levels quantified using ELISA. No previous exercise in the last 24 h (*previous fasting not specified*)	MA (endurance) vs UO: ↓ CRP; = IL-8, and TNF-α MA (endurance) vs UY: = IL-8, CRP, and TNF-α MA (sprint) vs UO: ↓ CRP; = IL-8, and TNF-α MA (sprint) vs UY: = IL-8, CRP, and TNF-α
Silva et al., 2016 [44]	*n* = 31 MA (15 high intensity; 73 (70–76) y and 16 moderate intensity; 69 (67–72.8) y, volleyball, basketball players, or endurance athletes) *n* = 15 UO; 70 (68–78) y (median (interquartile range))	MA (intense): male; 21 (10–30) y of training experience; at least some of them still competing; 23.3 (22.2–24.8) BMI MA (moderate): male; 10 (6.75–20) y of training experience; at least some of them still competing; 25.2 (23.8–26.6) BMI UO: male; 25.95 (22.6–28.1) BMI *(h/wk of training not reported)* (*IPAQ scores only graphically reported*) (median (interquartile range))	Fasting baseline circulating IL-6, IL-8, and IL-10 quantified using CBA. No previous exercise in the last 48 h	MA (intense) vs UO: = IL-6, IL-8, and IL-10 MA (moderate) vs UO: = IL-6, IL-8, and IL-10
Teixeira et al., 2021 [48]	*n* = 18 MA (judo, swimming, and athletics); 53.56 ± 9.25 y, *n* = 8 UO; 52.88 ± 5.64 y (mean ± SD)	MA: > 20 y of training experience; still competing; 25.62 ± 4.78 BMI UO: sedentary; 24.99 BMI (*h/wk of training, and sex not reported*) (mean ± SD)	Baseline circulating IL-8, IL-10, IL-12p70, and IL-17 quantified using multiplex immunoassay (*previous fasting and exercise not specified*)	MA vs UO: = IL-8, IL-10, IL-12p70, and IL-17

*BMI* body mass index, *CBA* cytometric bead array, *CRP* C-reactive protein, *DXA* dual-energy x-ray absorptiometry, *ECLIA* electrochemiluminescent immunoassay, *ELISA* enzyme-linked immunosorbent assay, *h* hours, *IL* interleukin, *IPAQ* International Physical Activity Questionnaire, *LIA* latex immunoturbidimetric assay, *MA* Master athletes, *SD* standard deviation, *SE* standard error, *TNF-α* tumor necrosis factor-α, *UO* untrained middle-aged and older controls, *UY* untrained young controls, *wk* week, *y* years

*Statistically significant difference from Master athletes

^a^Reported values were below reported lowest detectable level of the technique employed

A total of 649 subjects were documented in the included studies, including 349 Master athletes (trained subjects), 204 untrained middle-aged and older adults, and 96 young untrained adults. All studies reported data on the type of exercise or sport discipline performed, yet only two studies reported subgroup analyses on endurance and sprint/power sport athletes [37–39, 42]. Similarly, only three studies independently evaluated the impact of moderate and intense exercise lifestyle on inflammatory markers defined based on number of hours/days of training, distance covered in endurance efforts, or participation in competitive events [43–45].

The mean age of recruited Master athletes across all studies ranged between 45.5 and 74 years [42, 46], while untrained middle-aged and older adults ranged between 45.5 and 75 years [37, 46]. In contrast, the mean age of young adults ranged between 20 and 31.8 years [40, 47]. The sex of participants was reported in all but two studies [40, 48], with most of them recruiting only male subjects [37–39, 43–45, 47, 49–52], one study recruiting only female subjects [46], and three studies recruiting mixed cohorts [41, 42, 53]. Regarding training history, most studies reported average years of structured exercise experience that ranged between 14 and 53 years [45, 52], while three studies opted for reporting intervals [43, 48, 51] (i.e., > 30 years [51]). Only two studies reported average number of competitions per year (6.67–8.33/year) [37, 49], while most studies stated that athletes competed regularly or still competed, and two of them did not document participation in competitions [52, 53]. The number of hours of training per week was the most consistently reported metric when evaluating current training habits, with values that ranged between 5.4 and 12 h/wk [41, 50]. Alternatively, four authors documented running (33.8 to > 50 km/wk) or cycling (210 km/wk) distance covered per week [44, 47, 52, 53], while another two did not report data on training volume [48, 51]. Last, the physical activity habits of recruited non-trained controls were heterogeneously reported with six authors recruiting sedentary subjects [42, 43, 48, 50, 51, 53], four studies either reporting the number of steps per week [45, 46] or using physically activity questionnaires (International Physical Activity Questionnaire [IPAQ] [54]) [44, 52], two authors mentioning that control participants were recreationally active or physically active [37, 47], and another two studies not reporting any data on physical activity behaviors [40, 41, 49].

To evaluate inflammatory status, circulating levels of CRP were the most frequently reported inflammatory marker (nine studies) [40, 42, 43, 45–47, 51–53], followed by TNF-α [37, 40, 42, 43, 45, 46, 49, 52], and IL-6 [37, 40, 45, 46, 49, 51, 52] (eight studies each). Interleukin-10 data were reported in six studies [37, 40, 41, 44, 48–50], while IL-8 values were documented in four studies [40, 42, 44, 48], IL-17 in three studies [38, 40, 48], IL-15 in two studies [39, 40], and IL-1ra [40], IL-1β [40], IL-4 [40], IL-18 [38], and IL-12p70 [48] were only assessed in one study each. Regarding circulating levels of soluble receptors, sTNF-R1 was assessed in two studies [37, 52], sIL-6R was measured in one study [37], and sTNF-R2 in another study [52]. All studies reported the analytical technique used to quantify the inflammatory markers, with the most frequently used being enzyme-linked immunosorbent assay (ELISA).

### Risk of Bias Assessment

Results from the critical appraisal are presented in Table 3. Overall, two different studies were considered to be of high quality based on a risk of bias assessment (14.3%), while four (28.6%) had moderate quality, seven (50%) had low quality, and one remaining study (7.1%) had very low quality. A total of eight studies [37, 40–44, 47, 48, 50] reported inclusion criteria considered at recruitment, and surveyed training history and health status, while the remaining six studies [45, 46, 49, 51–53] either did not report inclusion criteria or recruited participants were insufficiently described. None of the included studies described in detail the study settings in terms of demographics, location, or period of time when the study was conducted. Most included studies defined lifelong exercise in a reliable manner and provided data on current training habits, while three studies did not report training habits of recruited athletes during the period of study [44, 51, 53]. Similarly, baseline inflammation was assessed through measuring at least two markers in all but three studies [47, 50, 53]. Although it is well established that certain dietary patterns can impact baseline inflammatory status in humans [55], only one included study attempted to describe dietary habits of recruited subjects (4-day food diary) [47]. Further, only five studies independently analyzed or adjusted at least one relevant confounder (i.e., percentage body fat mass, BMI, exercise type or intensity) when comparing levels of inflammatory markers across groups of study [37, 42, 43, 50, 51]. Regarding inflammatory outcomes, most studies declared assessing baseline serum or plasma levels of inflammatory markers in the fasting state using validated methods (i.e., ELISA), while four studies were unclear [40, 43, 48, 53]. Last, nine studies either did not report sample size calculations for primary study outcomes or reported unclear statistical analyses [40, 43–48, 51–53].

**Table 3:** Methodological quality assessment of included studies

Reference	Were the criteria for inclusion in the sample clearly defined?	Were the study subjects and the setting described in detail?	Was the exposure measured in a valid and reliable way?	Were objective, standard criteria used for measurement of the condition?	Were confounding factors identified?	Were strategies to deal with confounding factors stated?	Were the outcomes measured in a valid and reliable way?	Was appropriate statistical analysis used?	Methodological quality score (Y%)
Aguiar et al., 2020 [37–39]	Y	N	Y	Y	N	Y	Y	Y	High (75%)
Barbosa et al., 2021 [49]	N	N	Y	Y	N	N	Y	Y	Moderate (50%)
Girginer et al., 2019 [43]	Y	N	Y	Y	N	Y	N	N	Moderate (50%)
Gutierrez et al., 2021 [50]	Y	N	Y	N	N	Y	Y	Y	Moderate (62.5%)
Hayes et al., 2021 [51]	N	N	N	Y	N	Y	Y	N	Low (37.5%)
Lavin et al., 2020 [45]	N	N	Y	Y	N	N	Y	N	Low (37.5%)
Lavin et al., 2020 [46]	N	N	Y	Y	N	N	Y	N	Low (37.5%)
McKendry et al., 2019 [47]	Y	N	Y	N	Y	N	Y	N	Moderate (50%)
Mathur et a., 2013 [53]	N	N	N	N	N	N	N	N	Very low (0%)
Mikkelsen et al., 2013 [52]	N	N	Y	Y	N	N	Y	N	Low (37.5%)
Minuzzi et al., 2019 [40, 41]	Y	N	Y	Y	N	N	N	N	Low (37.5%)
Nickels et al., 2022 [42]	Y	N	Y	Y	N	Y	Y	Y	High (75%)
Silva et al., 2016 [44]	Y	N	N	Y	N	N	Y	N	Low (37.5%)
Teixeira et al., 2021 [48]	Y	N	Y	Y	N	N	N	N	Low (37.5%)

*N* negative answer (no), *Y* positive answer (yes)

### Quantitative Data Synthesis

**Fig. 2:**
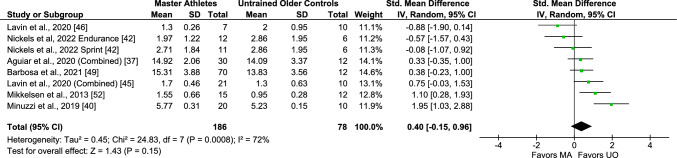
Meta-analysis of tumor necrosis factor-α between Master athletes (MA) and untrained middle-aged and older adult controls (UO). *CI* confidence interval, *SD* standard deviation

**Fig. 3:**
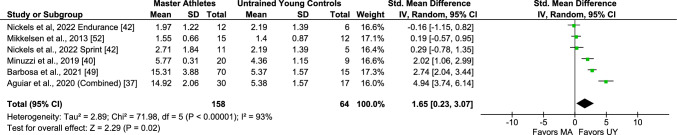
Meta-analysis of tumor necrosis factor-α between Master athletes (MA) and untrained young adult controls (UY). *CI* confidence interval, *SD* standard deviation

Differences in baseline circulating TNF-α levels between Master athletes and untrained middle-aged and older adults (presented as “untrained older controls” in figures) were reported in seven studies, including eight comparisons/arms (264 subjects). A non-significant effect estimate was observed from the pooled analysis of these studies (SMD 0.40, 95% CI − 0.15, 0.96, *I*^2^ 72%, *p* = 0.0008 [substantial heterogeneity]) (Fig. 2). Removing lower intensity exercise or non-endurance athletes did not significantly modify these findings (Figs. S1 and S2 of the ESM), while excluding female participants led to significantly higher levels in Master athletes (SMD 0.58, 95% CI 0.22, 0.93, moderate effect size, *I*^2^ 0%, *p* = 0.44) (Fig. S3 of the ESM). Regarding age-dependent differences, the meta-analysis of comparisons between Master athletes and untrained young adults (five studies including six comparisons/arms; 222 subjects) showed a significant effect estimate, resulting in lower levels in younger subjects compared with the Master athlete group (SMD 1.65, 95% CI 0.23, 3.07, large effect size, *I*^2^ 93%, *p* < 0.00001 [considerable heterogeneity]) (Fig. 3). This association remained significant after removing female subjects (Fig. S4 of the ESM), but not after removing non-endurance trained athletes (Fig. S5 of the ESM).

**Fig. 4:**
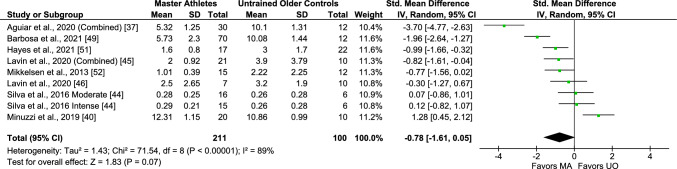
Meta-analysis of interleukin-6 between Master athletes (MA) and untrained middle-aged and older adult controls (*UO*)

**Fig. 5:**
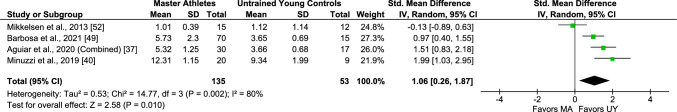
Meta-analysis of interleukin-6 between Master athletes (MA) and untrained young adult controls (*UY*)

Regarding IL-6, data on baseline circulating levels were reported in eight studies, including nine comparisons/arms (311 subjects). A non-significant effect estimate was observed in the pooled analysis of these studies (SMD − 0.78, 95% CI − 1.61, 0.5, *I*^2^ 89%, *p* < 0.00001 [considerable heterogeneity]) (Fig. 4), and the association remained non-significant when removing moderate-intensity exercise athletes (Fig. S6 of the ESM), although a trend towards lower levels in Master athletes was observed in both analyses. Subgroup analyses of these studies based on the type of exercise and participants’ sex showed significantly lower IL-6 levels in male Master athletes and endurance-trained Master athletes compared with middle-aged and older controls with large effect sizes, as shown in Figs. S7 and S8 of the ESM. Based on a meta-analysis findings, Master athletes still showed higher baseline levels of IL-6 when compared with untrained young adults as shown in Fig. 5 (four studies; 188 subjects) (SMD 1.06, 95% CI 0.26, 1.87, large effect size, *I*^2^ 80%, *p* < 0.002 (considerable heterogeneity); however, only a trend was shown after removing female participants (Fig. S9).

**Fig. 6:**
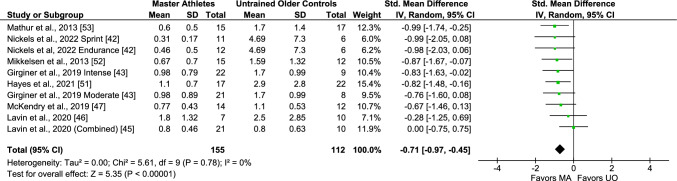
Meta-analysis of C-reactive protein between Master athletes (MA) and untrained middle-aged and older adult controls (*UO*)

**Fig. 7:**
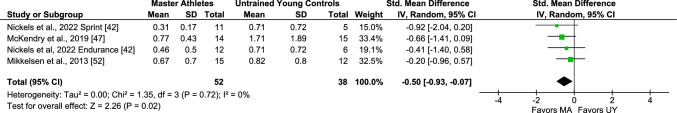
Meta-analysis of C-reactive protein between Master athletes (MA) and untrained young adults controls (UY)

The pooled analyses of eight studies (10 comparisons/arms; 267 subjects) reporting baseline circulating CRP values were homogeneous and indicated significantly lower CRP levels in Master athletes compared with untrained middle-aged and older adults (SMD − 0.71, 95% CI − 0.97, − 0.45, moderate effect size, *I*^2^ 0%, *p* = 0.78) (Fig. 6), which remained unchanged in all subgroup analyses (Figs. S10, S11, and S12 of the ESM). Only three studies (four comparisons/arms; 90 subjects) provided data on young untrained adults, and the pooled analysis revealed lower CRP levels in Master athletes compared with young adults (SMD − 0.50, 95% CI − 0.93, − 0.07, moderate effect size, I^2^ 0%, *p* = 0.72) (Fig. 7), which was not maintained after removing non-endurance athletes (Fig. S13 of the ESM). Last, only two studies reported CRP data as hs-CRP [43, 51], and thus no sub-analysis could be performed to evaluate the impact of low-sensitivity methods on these results.

**Fig. 8:**
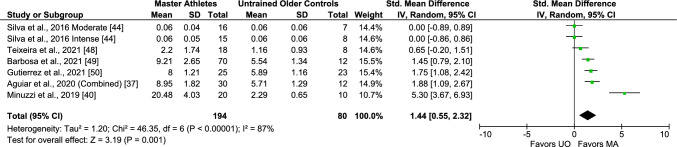
Meta-analysis of interleukin-10 between Master athletes (MA) and untrained middle-aged and older adult controls (UO)

A total of six studies including seven comparisons/arms (274 subjects) evaluating differences in IL-10 between Master athletes and untrained middle-aged and older adults were available for analyses. A significant pooled effect estimate resulted from the analyses denoting higher baseline IL-10 levels in the trained subjects (SMD 1.44, 95% CI 0.55, 2.32, large effect size, I^2^ 87%, *p* < 0.00001 [considerable heterogeneity]) (Fig. 8), which was maintained after removing lower-intensity exercise athletes and female participants (Figs. S14 and S15 of the ESM).

**Fig. 9:**
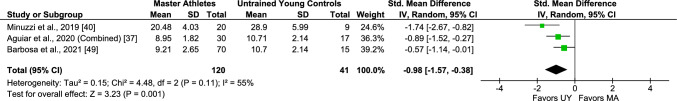
Meta-analysis of interleukin-10 between Master athletes (MA) and untrained young adult controls (UY)

Only three studies reported data on untrained young adults (161 subjects). Results from the meta-analysis of these studies indicated significantly lower IL-10 levels in Master athletes compared with the younger individuals (SMD − 0.98, 95% CI − 1.57, − 0.38, large effect size, *I*^2^ 55%, *p* = 0.11) [Fig. 9].

**Fig. 10:**
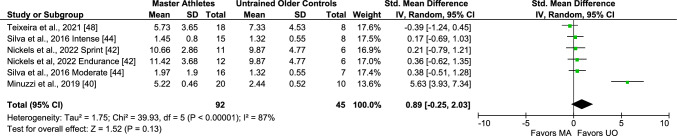
Meta-analysis of interleukin-8 between Master athletes (MA) and untrained middle-aged and older adult controls (UO)

**Fig. 11:**
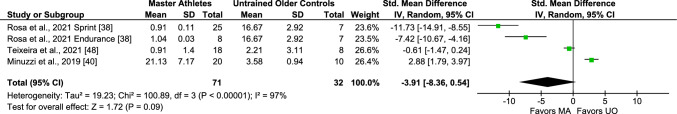
Meta-analysis of interleukin-17 between Master athletes (MA) and untrained middle-aged and older adult controls (UO). Rosa et al. [38] participants are included in the Aguiar et al. population [37] (characteristics presented in Table 2)

Regarding IL-8, four studies including six comparisons/arms (137 subjects) involved Master athletes and untrained middle-aged and older adults. The pooled analysis of these studies revealed a non-significant effect estimate (SMD 0.89, 95% CI − 0.25, 2.03, *I*^2^ 87%, *p* < 0.0001 [considerable heterogeneity]) (Fig. 10), indicating similar IL-8 levels between groups, which remained unchanged after removing lower-intensity exercise athletes (Fig. S16 of the ESM). Last, only three studies (four comparisons/arms; 103 subjects) were available for the IL-17 meta-analysis. Findings from pooled analyses showed similar IL-17 levels in Master athletes and untrained middle-aged and older adults (SMD − 3.91, 95% CI − 8.36, 0.54, *I*^2^ 97%, *p* < 0.00001 [considerable heterogeneity]) (Fig. 11). No pooled analyses involving young adults, or different subgroup analyses were possible for IL-8 or IL-17.

## Discussion

In the present work, we systematically reviewed the available scientific studies comparing healthy Master athletes, untrained middle-aged and older adults, and untrained young subjects to shine light upon the impact of lifelong exercise on low-grade inflammation associated with aging. The main findings from the present systematic review and meta-analysis suggest that lifelong exercise, defined by at least 10 years of consistent structured training experience, might be associated with decreased baseline levels of CRP and increased levels of anti-inflammatory IL-10 compared with age-matched untrained subjects. Further, CRP and IL-10 findings were shown to be independent of exercise intensity and were not impacted by the exclusion of female participants, with CRP results being also independent of type of exercise. In the same vein, a trend towards decreased IL-6 (typically considered a proinflammatory cytokine) was observed in the main analyses, and rendered statistically significant when sub-analyzing male subjects and endurance-trained athletes. Altogether, these results suggest potential benefits of lifelong structured exercise patterns on chronic systemic inflammation associated with aging. However, analyses comparing levels of several cytokines (IL-6, IL-10, TNF-α) in Master athletes and young individuals indicated that lifelong-trained middle-aged and older adults might still present a more proinflammatory profile than non-trained young subjects, with the exception of CRP analyses, which were based on a limited number of studies. Last, no effect of lifelong exercise on IL-8 and IL-17 levels was found, and TNF-α analyses yielded mixed results with limited evidence, suggesting increased levels in male Master athletes compared with age-matched non-trained subjects.

### Interleukin-6

Interleukin-6 has been considered “a cytokine for gerontologists” for decades [56], and is regarded as the most informative marker of inflammaging in older adults [16]. These statements are supported by studies documenting linear increases in baseline IL-6 levels with aging [57, 58], and by large prospective studies identifying IL-6 as a robust independent predictor of all-cause mortality in older adults [59–62]. Compared to physical inactivity, adherence to physical activity guidelines [63], and participation in long-term aerobic exercise programs have been shown to be effective to for attenuating increases in circulating IL-6 levels [22]. Based on the present meta-analysis, only a trend towards increased IL-6 levels was found in untrained compared with lifelong-trained middle-aged and older adults, while significant decrements were observed in male and endurance-trained Master athletes. Divergent results based on type of training are not surprising as reflected in meta-analyses concluding beneficial effects of long-term aerobic [22] but not resistance training [21] on IL-6 levels in older adults. However, sex-based differences in age-related increases in baseline IL-6 remain largely unexplored [64], and only four studies recruiting female subjects were identified through the search strategy, which highlights a need for further research conducted in female populations. Notably, Master athletes were shown to present higher levels than young non-trained adults, albeit a limited number of studies were pooled in the main analyses. These results are aligned with those obtained in a comprehensive analysis conducted in physically active (non-competitive) middle-aged and older cyclists, who were shown to present notably attenuated baseline IL-6 levels compared with age-matched inactive peers, yet levels were even lower in a further comparison group consisting of healthy young adults [65]. Altogether, these findings suggest that while Master athletes might benefit from attenuated baseline IL-6 levels compared to non-trained middle-aged and older adults, lifelong exercise might not completely counteract age-related increases in circulating IL-6.

It is worth mentioning that acute exercise is demonstrated to induce local skeletal muscle production of IL-6, which can translate to over 100-fold increases in plasma levels of this myokine [66]. Contrary to chronic IL-6 increases, this acute short-lived response is eminently anti-inflammatory and is characterized by IL-6-mediated increments in anti-inflammatory IL-10 and IL-1ra expression [67]. With the exception of Barbosa et al.'s study [49], all studies pooled in the present meta-analysis recommended refraining from physical exercise at least 24 h before blood sampling, meaning influence of exercise-mediated acute increases in IL-6 may be reasonably disregarded in the analyses.

### C-Reactive Protein

C-reactive protein is another key component of the inflammaging framework which has been shown to predict mortality in middle-aged and older adults [68, 69]. Hepatic synthesis of CRP is largely triggered in response to IL-6 signaling [70], which helps explain increases during post-exercise recovery following the initial exercise-induced IL-6 response [71]. As commented, long-term exercise training has been reported to attenuate baseline circulating CRP levels in middle-aged and older adults [21, 22]. Consistent with this our findings support a role of lifelong exercise training in reducing CRP levels in Master athletes compared with untrained middle-aged and older adults, which was shown to be independent of exercise type and intensity, and was maintained in analyses including either mixed cohorts or male participants only. Lower CRP values in Master athletes compared with untrained young subjects were also observed in the analyses; however, only three studies were pooled, and the results were not statistically significant in subgroup analyses. Altogether, these findings support a consistent role of lifelong exercise in attenuating age-related increases in this marker.

### Tumor Necrosis Factor-α

Parallel to IL-6 and CRP, TNF-α is generally considered a proinflammatory cytokine upregulated with aging and associated with all-cause mortality in healthy older adults [72], albeit most evidence arises from studies conducted in fragile individuals [73–76]. Further, some preclinical research exploring TNF-α antagonists has supported a causative role of aging in the onset and severity of stroke mediated by heightened TNF-α levels [77]. In contrast to the above mentioned effects of exercise on other inflammatory markers, some quantitative analyses of the literature have failed to identify significant attenuating effects of exercise training on TNF-α levels in older adults [21, 78], which is in line with our analyses showing no clear impact of lifelong exercise on circulating TNF-α concentrations levels in Master athletes. Untrained and trained middle-aged and older adults had similar TNF-α levels as shown in the main analyses, yet significantly higher levels were apparent in male Master athletes. Conversely, young adults had lower TNF-α than Master athletes, but this depended on the type of exercise performed. Increments in baseline TNF-α might be particularly relevant to Master athletes as TNF-α-mediated activation of nuclear factor-κB has been posited to contribute to skeletal muscle anabolic resistance associated with aging [79], which in turn might compromise recovery from exercise-induced muscle damage [80].

Tumor necrosis factor-α is not immediately released in response to exercise, and changes in adiposity have been proposed to be a major contributor to increments in circulating TNF-α levels associated with aging [81, 82]. It might be thus speculated that differences in body composition may have impacted our results regarding TNF-α analyses. However, most studies included in these analyses reported higher BMI [37, 42, 45, 46, 49, 52], and increased percentage body fat mass [37, 42, 45, 46, 49] in non-trained compared with trained middle-aged and older adults, with some of them documenting statistically significant differences [37, 42, 45, 46, 49]. In particular, Aguiar et al. documented similar TNF-α levels in Master athletes and untrained middle-aged and older adults, and reported an age-adjusted direct association between percentage body fat mass and TNF-α levels in non-trained subjects [37]. However, this association was not significant in Master athletes when analyzed separately, thus suggesting that body adiposity could not explain TNF-α levels in these subjects [37]. Conversely, authors argued that observed differences in other markers (IL-6, sIL-6R, sTNF-R, and IL-10) between trained and untrained middle-aged and older adults might have been caused by increased adiposity in non-trained participants [37].

### Interleukin-10

Interleukin-10 is arguably the most widely studied anti-inflammatory cytokine in humans [83]. It is produced by most leukocytes, and is responsible for repressing macrophage activation and the subsequent release of proinflammatory cytokines, such as IL-6 and TNF-α [83]. Baseline IL-10 levels have been shown to indirectly correlate with aging in some studies [84], but direct associations have been described in other studies [85, 86]. Age-related increases might be considered as a compensatory mechanism aimed to help dampen proinflammatory cytokine responses. Notably, some authors have proposed high IL-10 production capacity as a marker of human longevity in long-lived individuals [87]. In the present meta-analysis, we observed a large effect size indicating higher IL-10 in Master athletes compared with untrained middle-aged and older adults, which might support the notion of elevated IL-10 levels as a feature of successful aging. Conversely, the pooled analysis of a limited number of studies suggested that Master athletes still present lower IL-10 than young untrained adults, which is overall aligned with increases in IL-6 and TNF-α shown in analyses, and with the role of IL-10 in attenuating the release and activity of these proinflammatory cytokines.

### Other Inflammation-Related Markers

No effect of lifelong exercise on IL-8 and IL-17 levels was found in the present meta-analysis, and no analyses could be performed on different cytokines or soluble receptors. While comprehensively evaluating inflammatory marker panels is important to delineate low-grade inflammation [40], reviewed studies often failed to provide a rationale for analyzing specific cytokines (i.e., IL-8 [42, 44]). Supplementing IL-10 analyses with other anti-inflammatory cytokine assessments (i.e., IL-1ra and IL-4) might be of value when evaluating potential compensatory anti-inflammatory mechanisms in trained middle-aged and older subjects. Additionally, analyses of leukocyte populations might shine light upon potential sources of chronic low-grade inflammation. In this sense, Silva et al., reported an attenuated shift from naïve T-cells towards memory T cells in aerobic trained Master athletes, which is aligned with different trials exploring the impact of exercise on T-cell compartments [65, 88], and supports the role of immune senescence as a contributor to low-grade inflammation associated with aging.

### Limitations and Future Research Outlook

While this is the first systematic analysis on this topic, the findings reported here should not be considered conclusive but should encourage further investigation. A total of 17 reports were identified through search in electronic databases, yet these included a modest total number of participants (*n* = 649). Further, only two studies were considered to have “high” methodological quality based on risk of bias assessment, yet even these two studies failed to clearly define study settings (i.e., demographics, time, and location), or report data on dietary intakes [37, 42]. This may be particularly relevant as nutrition is the most important modifiable factor contributing to inflammatory profiles in humans along with exercise. While comprehensive evaluations of dietary intakes in lifelong exercisers are challenging, further studies might benefit from surveying the use of anti-inflammatory dietary supplements [89] as well as potential adherence to specific dietary patterns (i.e., vegan/vegetarian diets [90]) in recruited participants. Other relevant methodological uncertainties consistently observed in identified studies include insufficient characterization of physical activity habits in control group participants, heterogeneous reporting of body composition outcomes, and a lack of statistical adjustment of relevant confounders in analyses of inflammatory markers. In addition to quality assessment findings, a major limitation of the available evidence consists of the cross-sectional nature of baseline cytokine analyses. Repeated testing in longitudinal analyses might help these studies to overcome short-term variations in explored markers. These studies would also benefit from using robust and high sensitive analytical methods, such as multiplex array platforms, to better capture low-grade inflammation through the simultaneous analysis of multiple biomarkers [91].

Several reviewed studies reported measurements of body adiposity and documented increased percentage body fat mass in untrained compared with trained middle-aged and older adults [42, 43, 45–47, 49]; however, these data were often not considered in analyses of inflammatory markers, making it difficult to evaluate the impact of adiposity on findings related to low-grade inflammation. In addition to the Aguiar et al. study [37], only two different studies explored associations between percentage body fat mass and levels of proinflammatory and anti-inflammatory markers [50, 51]. Specifically, Hayes et al. observed that baseline IL-6 and CRP levels were directly associated with percentage body fat, while controlling for body fat was shown to attenuate CRP differences between Master athletes and untrained age-matched peers [51]. In alignment with Aguiar et al., [37] Gutierrez et al. also documented a negative association between IL-10 levels and percentage body fat adjusted for BMI and age in both Master athletes and untrained middle-aged and older adults [50]. Overall, differences in body composition might partially drive the effects of lifelong exercise on attenuating low-grade inflammation in different inflammatory markers, but the available evidence is limited. Further studies should carefully control for lean and fat mass to better understand age-related variations in inflammatory marker levels and changes induced in response to long-term exercise. This may be particularly relevant in studies recruiting female subjects, who are typically characterized by higher adiposity compared with their male counterparts.

Our research is not exempt from limitations. We observed considerable heterogeneity in most pooled analyses, yet identifying sources of heterogeneity is challenging owing to the limited number of available comparisons. A potential source of heterogeneity might include within-group age differences in some pooled analyses (i.e., the mean age difference between young untrained adults and Master athletes was ≈20 years greater in the Mikkelsen et al. study [52] than in the Nickels et al. study [42]). While we separately analyzed studies evaluating sprint and endurance athletes as well as studies including athletes training at different intensities, clearly delineating the impact of exercise type and intensity remains challenging as pooled analyses still included endurance-trained athletes from different sport disciplines (i.e., swimming, athletics), and with heterogeneous training frequencies/volumes. Further research is necessary to better comprehend how varying training frequencies, volumes, and exercise types affect inflammatory outcomes with aging. This is especially true for resistance training, which is clearly neglected in the available literature. These studies would benefit from recruiting participants across different age ranges to help clarify whether each year of aging has a comparable effect on levels of discussed inflammatory markers, and how exercise contributes to counteract low-grade inflammation in individuals of different ages and different years of training experience. In addition, only four studies included women in the cohorts of study, meaning our results might not be able to be extrapolated to female populations. Further studies recruiting female participants are needed to help elucidate sex-based differences in inflammatory markers in response to exercise and evaluate the impact of menopause in female Master athletes. Studies recruiting female participants of reproductive age should aim at controlling menstrual cycles at the sample collection to support the validity of their findings [92]. While we considered analyzing data on additional markers of low-grade inflammation associated with aging, namely interferon-γ [93], we could not find any study reporting quantifiable levels, which made unfeasible such analyses. Last, we limited our search to publications written in English, so it remains uncertain how records in other languages might affect our findings. Despite the abovementioned limitations, our results support the prescription of exercise as a valuable strategy to attenuate low-grade inflammation associated with aging, which in turn might have positive repercussions on the incidence of age-related adverse events linked to inflammatory conditions.

## Conclusions

Based on the present meta-analysis, Master athletes may exhibit attenuated baseline circulating levels of CRP and, potentially, IL-6, along with a higher level of anti-inflammatory IL-10 compared with age-matched non-trained healthy subjects, which overall suggests a role of lifelong structured exercise patterns in counteracting low-grade inflammation associated with aging. However, the available evidence appears to indicate that untrained young adults still present with lower resting levels of IL-6 and TNF-α in combination with higher IL-10 compared with lifelong-trained middle-aged and older adults, which denotes that training per se might not be sufficient to completely prevent chronic systemic inflammation in middle-aged and older adults. These findings should be considered with caution as most reviewed studies had low-to-moderate quality, and considerable heterogeneity was observed in several pooled analyses, some of them including a limited number of studies. Further studies recruiting Master athletes might benefit from conducting longitudinal testing of inflammatory biomarker panels using sensitive methods, analyzing different inflammatory indicators such as leukocyte counts and phenotypes, and considering the potential impact that relevant confounders - mainly nutrition, but also body composition, type of exercise, and sex - may have on reported findings to deepen our understanding of low-grade inflammation associated with aging.

## Supplementary Information

Below is the link to the electronic supplementary material.

Supplementary file1 (DOCX 17 KB)

Supplementary file2 (DOCX 64 KB)

Supplementary file3 (DOCX 9008 KB)

## Declarations

### Availability of Data and Material

All data used in the analyses are available on request to the corresponding author.
