# Supplementary material for: Does Lifelong Exercise Counteract Low-Grade Inflammation Associated with Aging? A Systematic Review and Meta-Analysis
Source: Sports Med. 2025 Jan 10;55(3):675–96. doi: 10.1007/s40279-024-02152-8 (PMC11985631; doi:10.1007/s40279-024-02152-8)
Supplement: Supplementary file 1 — Supplementary file1 (DOCX 17 KB) [file 40279_2024_2152_MOESM1_ESM.docx]

**Supplementary 1**

**Table S1. Search Strategy**

| Database | Search String | Nº of records retrieved | Date of last search update |
| --- | --- | --- | --- |
| PubMed/MEDLINE | ("life-long exercise"[Title/Abstract] OR "life-long physical activity"[Title/Abstract] OR "lifelong exercise"[Title/Abstract] OR "lifelong physical activity"[Title/Abstract] OR "master athlete"[Title/Abstract] OR "aging athlete"[Title/Abstract] OR "middle-aged athlete"[Title/Abstract] OR "lifelong endurance"[Title/Abstract] OR "life-long endurance"[Title/Abstract] OR "lifelong resistance"[Title/Abstract] OR "life-long resistance"[Title/Abstract]) AND ("inflammation"[MeSH Terms] OR "Inflammation"[Title/Abstract] OR "inflammatory"[Title/Abstract] OR "pro-inflammatory"[Title/Abstract] OR "anti-inflammatory"[Title/Abstract] OR "cytokine"[Title/Abstract] OR "telomere"[Title/Abstract] OR "recover*"[Title/Abstract]) | 63 | Sep. 23^rd^, 2024 |
| CENTRAL | ("life-long exercis*" OR "life-long physical* activ*" OR "lifelong exercis*" OR "lifelong physical* active*" OR "master athlet*" OR "aging athlet*" OR "middle-aged athlet*" OR "lifelong endurance" OR "life-long endurance" OR "lifelong resistance" OR "life-long resistance") AND ("inflamm*" OR "pro-inflamm*" OR "anti-inflamm*" OR "cytokine?" OR "telomere" OR "recover*") in Title Abstract Keyword | 5 | Sep. 23^rd^, 2024 |
| EMBASE | ("life-long exercis*" OR "life-long physical* activ*" OR "lifelong exercis*" OR "lifelong physical* active*" OR "master athlet*" OR "aging athlet*" OR "middle-aged athlet*" OR "lifelong endurance" OR "life-long endurance" OR "lifelong resistance" OR "life-long resistance") AND ("inflamm*" OR "pro-inflamm*" OR "anti-inflamm*" OR "cytokine?" OR "telomere" OR "recover*") | 133 | Sep. 23^rd^, 2024 |
| Web of Science | TS=("life-long exercis*" OR "life-long physical* activ*" OR "lifelong exercis*" OR "lifelong physical* active*" OR "master athlet*" OR "aging athlet*" OR "middle-aged athlet*" OR "lifelong endurance" OR "life-long endurance" OR "lifelong resistance" OR "life-long resistance") AND ("inflamm*" OR "pro-inflamm*" OR "anti-inflamm*" OR "cytokine?" OR "telomere" OR "recover*") | 149 | Sep. 23^rd^, 2024 |
| SPORTdiscus | ("life-long exercis*" OR "life-long physical* activ*" OR "lifelong exercis*" OR "lifelong physical* active*" OR "master athlet*" OR "aging athlet*" OR "middle-aged athlet*" OR "lifelong endurance" OR "life-long endurance" OR "lifelong resistance" OR "life-long resistance") AND ("inflamm*" OR "pro-inflamm*" OR "anti-inflamm*" OR "cytokine?" OR "telomere" OR "recover*") | 657 | Sep. 23^rd^, 2024 |
| Scopus | ("life-long exercis*" OR "life-long physical* activ*" OR "lifelong exercis*" OR "lifelong physical* active*" OR "master athlet*" OR "aging athlet*" OR "middle-aged athlet*" OR "lifelong endurance" OR "life-long endurance" OR "lifelong resistance" OR "life-long resistance") AND ("inflamm*" OR "pro-inflamm*" OR "anti-inflamm*" OR "cytokine?" OR "telomere" OR "recover*") | 138 | Sep. 23^rd^, 2024 |
