# Supplementary material for: Does Lifelong Exercise Counteract Low-Grade Inflammation Associated with Aging? A Systematic Review and Meta-Analysis
Source: Sports Med. 2025 Jan 10;55(3):675–96. doi: 10.1007/s40279-024-02152-8 (PMC11985631; doi:10.1007/s40279-024-02152-8)
Supplement: Supplementary file 2 — Supplementary file2 (DOCX 64 KB) [file 40279_2024_2152_MOESM2_ESM.docx]

**Supplementary 2**

**Table S2 Reasons for Exclusion**

| Reason | Number |
| --- | --- |
| Mean years of participation in structured exercise/training was lower than 10 or was not stated | (1) |
| No circulating levels of markers of inflammatory status were reported, or analyses yielded non-quantifiable levels, thus impeding comparisons | (2) |
| Mean age of participants differed from inclusion criteria | (3) |
| Only recruited former master athletes or non-athlete exercisers | (4) |
| Lack of untrained control group | (5) |

**Table S3 Excluded Studies**

| Reference | Reason |
| --- | --- |
| Balan et al., 2020 ^1^ | (1) |
| Balan et al., 2021 ^2^ | (1) |
| Bhella et al., 2014 ^3^ | (2) |
| Bolotta et al., 2020 ^4^ | (2) |
| Bunprajun et al., 2013 ^5^ | (2) |
| Chapman et al., 2020 ^6^ | (2) |
| De Sanctis et al., 2021 ^7^ | (2) |
| Dethlefsen et al., 2018 ^8^ | (1,2) |
| Duggal et al., 2018 ^9^ | (4) |
| Fernandes et al., 2019 ^10^ | (2) |
| Frandsen et al., 2022 ^11^ | (1,2) |
| Herbert et al., 2017 ^12^ | (2) |
| Kamijo et al., 2009 ^13^ | (1,3) |
| Koltai et a., 2018 ^14^ | (2) |
| Laine et al., 2015 ^15^ | (4) |
| Leite et al., 2023 ^16^ | (2) |
| Lendvorsky et al., 2021 ^17^ | (2) |
| Mackey et al., 2014 ^18^ | (2) |
| Majerczak et a., 2019 ^19^ | (4) |
| Markus et al., 2022 ^20^ | (2,4) |
| Martin et al., 2015 ^21^ | (1,2) |
| Mikkelsen et al., 2017 ^22^ | (2) |
| Minuzzi et al., 2018 ^23^ | (2) |
| Minuzzi et al., 2024 ^24^ | (5) |
| Perkins et a., 2024 ^25^ | (2) |
| Rivier et al., 1994 ^26^ | (2) |
| Sailani et al., 2019 ^27^ | (1) |
| Yasar et al., 2021 ^28^ | (1) |

1. Balan, E.; De Groote, E.; Bouillon, M.; Viceconte, N.; Mahieu, M.; Naslain, D.; Nielens, H.; Decottignies, A.; Deldicque, L., No effect of the endurance training status on senescence despite reduced inflammation in skeletal muscle of older individuals. *American Journal of Physiology-Endocrinology and Metabolism* **2020,** *319* (2), E447-E454.

2. Balan, E.; Diman, A.; Everard, A.; Nielens, H.; Decottignies, A.; Deldicque, L., Endurance training alleviates MCP-1 and TERRA accumulation at old age in human skeletal muscle. *Experimental Gerontology* **2021,** *153*.

3. Bhella, P. S.; Hastings, J. L.; Fujimoto, N.; Shibata, S.; Carrick-Ranson, G.; Palmer, M. D.; Boyd, K. N.; Adams-Huet, B.; Levine, B. D., Impact of Lifelong Exercise "Dose" on Left Ventricular Compliance and Distensibility. *Journal of the American College of Cardiology* **2014,** *64* (12), 1257-1266.

4. Bolotta, A.; Filardo, G.; Abruzzo, P. M.; Astolfi, A.; De Sanctis, P.; Di Martino, A.; Hofer, C.; Indio, V.; Kern, H.; Lofler, S.; Marcacci, M.; Zampieri, S.; Marini, M.; Zucchini, C., Skeletal Muscle Gene Expression in Long-Term Endurance and Resistance Trained Elderly. *Int J Mol Sci* **2020,** *21* (11).

5. Bunprajun, T.; Henriksen, T. I.; Scheele, C.; Pedersen, B. K.; Green, C. J., Lifelong Physical Activity Prevents Aging-Associated Insulin Resistance in Human Skeletal Muscle Myotubes via Increased Glucose Transporter Expression. *PLoS ONE* **2013,** *8* (6).

6. Chapman, M. A.; Arif, M.; Emanuelsson, E. B.; Reitzner, S. M.; Lindholm, M. E.; Mardinoglu, A.; Sundberg, C. J., Skeletal Muscle Transcriptomic Comparison between Long-Term Trained and Untrained Men and Women. *Cell Reports* **2020,** *31* (12).

7. De Sanctis, P.; Filardo, G.; Abruzzo, P. M.; Astolfi, A.; Bolotta, A.; Indio, V.; Di Martino, A.; Hofer, C.; Kern, H.; Loefler, S.; Marcacci, M.; Marini, M.; Zampieri, S.; Zucchini, C., Non-Coding RNAs in the Transcriptional Network That Differentiates Skeletal Muscles of Sedentary from Long-Term Endurance- and Resistance-Trained Elderly. *International Journal of Molecular Sciences* **2021,** *22* (4).

8. Dethlefsen, M. M.; Halling, J. F.; Møller, H. D.; Plomgaard, P.; Regenberg, B.; Ringholm, S.; Pilegaard, H., Regulation of apoptosis and autophagy in mouse and human skeletal muscle with aging and lifelong exercise training. *Experimental gerontology* **2018,** *111*, 141-153.

9. Duggal, N. A.; Pollock, R. D.; Lazarus, N. R.; Harridge, S.; Lord, J. M., Major features of immunesenescence, including reduced thymic output, are ameliorated by high levels of physical activity in adulthood. *Aging cell* **2018,** *17* (2), e12750.

10. Fernandes, J. F. T.; Lamb, K. L.; Twist, C., Exercise-Induced Muscle Damage and Recovery in Young and Middle-Aged Males with Different Resistance Training Experience. *Sports (2075-4663)* **2019,** *7* (6), 132.

11. Frandsen, J.; Sahl, R. E.; Rømer, T.; Hansen, M. T.; Nielsen, A. B.; Lie‐Olesen, M. M.; Rasmusen, H. K.; Søgaard, D.; Ingersen, A.; Rosenkilde, M.; Westerterp, K.; Holst, J. J.; Andersen, J. L.; Markowski, A. R.; Blachnio‐Zabielska, A.; Clemmensen, C.; Sacchetti, M.; Cataldo, A.; Traina, M.; Larsen, S., Extreme duration exercise affects old and younger men differently. *Acta Physiologica* **2022,** *235* (3), 1-18.

12. Herbert, P.; Hayes, L. D.; Sculthorpe, N.; Grace, F. M., High-intensity interval training (HIIT) increases insulin-like growth factor-I (IGF-I) in sedentary aging men but not masters' athletes: an observational study. *Aging Male* **2017,** *20* (1), 54-59.

13. Kamijo, T.; Murakami, M., Regular Physical Exercise Improves Physical Motor Functions and Biochemical Markers in Middle-Age and Elderly Women. *Journal of Physical Activity & Health* **2009,** *6* (1), 55-62.

14. Koltai, E.; Bori, Z.; Osvath, P.; Ihasz, F.; Peter, S.; Toth, G.; Degens, H.; Rittweger, J.; Boldogh, I.; Radak, Z., Master athletes have higher miR-7, SIRT3 and SOD2 expression in skeletal muscle than age-matched sedentary controls. *Redox Biology* **2018,** *19*, 46-51.

15. Laine, M. K.; Eriksson, J. G.; Kujala, U. M.; Raj, R.; Kaprio, J.; Bäckmand, H. M.; Peltonen, M.; Sarna, S., Effect of Intensive Exercise in Early Adult Life on Telomere Length in Later Life in Men. *Journal of Sports Science & Medicine* **2015,** *14* (2), 239-245.

16. Leite, P. L. d. A.; Maciel, L. A.; Santos, P. A.; Barbosa, L. P.; Gutierrez, S. D.; Corrêa, H. d. L.; Deus, L. A. d.; Araújo, M. C.; Aguiar, S. d. S.; Rosa, T. D. S.; Lewis, J. E.; Simões, H. G., Higher sirt1 is associated with a better body composition in master sprinters and untrained peers. *European journal of sport science* **2023,** *23* (7), 1251-1258.

17. Lendvorsky, L.; Smolkova, B.; Buocikova, V.; Wachsmannova, L.; Bielik, V., Global DNA methylation and physical fitness of elderly athletes with lifelong endurance activity. *Journal of Human Sport & Exercise* **2021,** *16* (4), 929-940.

18. Mackey, A. L.; Karlsen, A.; Couppé, C.; Mikkelsen, U. R.; Nielsen, R. H.; Magnusson, S. P.; Kjaer, M., Differential satellite cell density of type I and II fibres with lifelong endurance running in old men. *Acta Physiologica* **2014,** *210* (3), 612-627.

19. Majerczak, J.; Grandys, M.; Frołow, M.; Szkutnik, Z.; Zakrzewska, A.; Ni_Zankowski, R.; Duda, K.; Chlopicki, S.; Zoladz, J. A., Age-dependent impairment in endothelial function and arterial stiffness in former high class male athletes is no different to that in men with no history of physical training. *Journal of the American Heart Association* **2019,** *8* (18), 1-14.

20. Markus, I.; Constantini, K.; Goldstein, N.; Amedi, R.; Bornstein, Y.; Stolkovsky, Y.; Vidal, M.; Lev-Ari, S.; Balaban, R.; Leibou, S.; Blumenfeld-Katzir, T.; Ben-Eliezer, N.; Peled, D.; Assaf, Y.; Jensen, D.; Constantini, N.; Dubnov-Raz, G.; Halperin, I.; Gepner, Y., Age Differences in Recovery Rate Following an Aerobic-Based Exercise Protocol Inducing Muscle Damage Among Amateur, Male Athletes. *Front Physiol* **2022,** *13*, 916924.

21. Martin, T. G.; Pata, R. W.; D'Addario, J.; Yuknis, L.; Kingston, R.; Feinn, R., Impact of age on haematological markers pre- and post-marathon running. *Journal of Sports Sciences* **2015,** *33* (19), 1988-1997.

22. Mikkelsen, U. R.; Agergaard, J.; Couppé, C.; Grosset, J. F.; Karlsen, A.; Magnusson, S. P.; Schjerling, P.; Kjaer, M.; Mackey, A. L., Skeletal muscle morphology and regulatory signalling in endurance-trained and sedentary individuals: The influence of ageing. *Experimental Gerontology* **2017,** *93*, 54-67.

23. Minuzzi, L. G.; Rama, L.; Chupel, M. U.; Rosado, F.; dos Santos, J. V.; Simpson, R.; Martinho, A.; Paiva, A.; Teixeira, A. M., Effects of lifelong training on senescence and mobilization of T lymphocytes in response to acute exercise. *Exercise Immunology Review* **2018,** *24*, 34-46.

24. Minuzzi, L. G.; Ferrauti, A.; Chupel, M. U.; Hacker, S.; Weyh, C.; Valenzuela, P. L.; Lucia, A.; Krüger, K.; Reichel, T., Acute Inflammatory Response to Eccentric Exercise in Young and Master Resistance-trained Athletes. *Int J Sports Med* **2024**.

25. Perkins, R. K.; Lavin, K. M.; Raue, U.; Jemiolo, B.; Trappe, S. W.; Trappe, T. A., Effects of aging and lifelong aerobic exercise on expression of innate immune components in skeletal muscle of women. *J Appl Physiol (1985)* **2024,** *136* (3), 482-491.

26. Rivier, A.; Pene, J.; Chanez, P.; Anselme, F.; Caillaud, C.; Prefaut, C.; Godard, P.; Bousquet, J., Release of cytokines by blood monocytes during strenuous exercise. *International Journal of Sports Medicine* **1994,** *15* (4), 192-198.

27. Sailani, M. R.; Halling, J. F.; Møller, H. D.; Lee, H.; Plomgaard, P.; Pilegaard, H.; Snyder, M. P.; Regenberg, B., Lifelong physical activity is associated with promoter hypomethylation of genes involved in metabolism, myogenesis, contractile properties and oxidative stress resistance in aged human skeletal muscle. *Scientific reports* **2019,** *9* (1), 3272.

28. Yasar, Z.; Elliott, B. T.; Kyriakidou, Y.; Nwokoma, C. T.; Postlethwaite, R. D.; Gaffney, C. J.; Dewhurst, S.; Hayes, L. D., Sprint interval training (SIT) reduces serum epidermal growth factor (EGF), but not other inflammatory cytokines in trained older men. *Eur J Appl Physiol* **2021,** *121* (7), 1909-1919.
