# Supplementary material for: Does Lifelong Exercise Counteract Low-Grade Inflammation Associated with Aging? A Systematic Review and Meta-Analysis
Source: Sports Med. 2025 Jan 10;55(3):675–96. doi: 10.1007/s40279-024-02152-8 (PMC11985631; doi:10.1007/s40279-024-02152-8)
Supplement: Supplementary file 3 — Supplementary file3 (DOCX 9008 KB) [file 40279_2024_2152_MOESM3_ESM.docx]

**Supplementary 3**


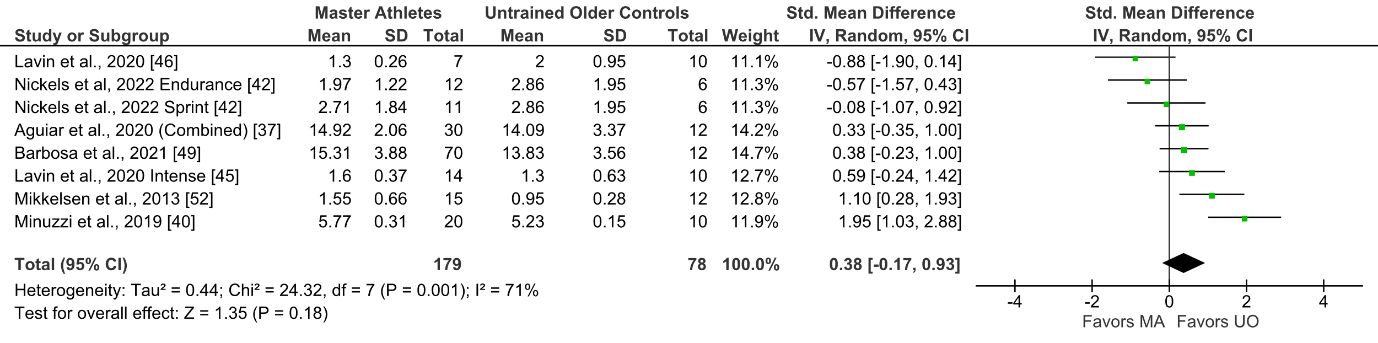


**Figure S1.** Subgroup analysis of TNF-α between master athletes and untrained middle-aged and older adults excluding moderate intensity athletes. MA, master athletes; UO, untrained middle-aged and older controls


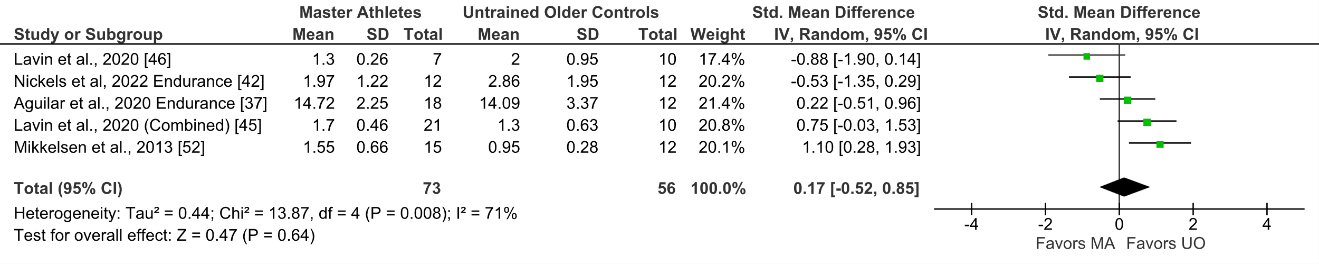


**Figure S2**. Subgroup analysis of TNF-α between master athletes and untrained middle-aged and older adults excluding non-endurance exercise athletes. MA, master athletes; UO, untrained middle-aged and older controls


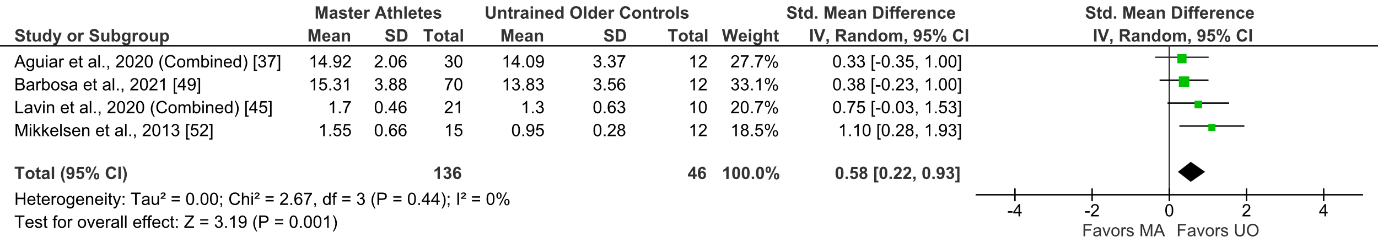


**Figure S3**. Subgroup analysis of TNF-α between master athletes and untrained middle-aged and older adults excluding female subjects. MA, master athletes; UO, untrained middle-aged and older controls
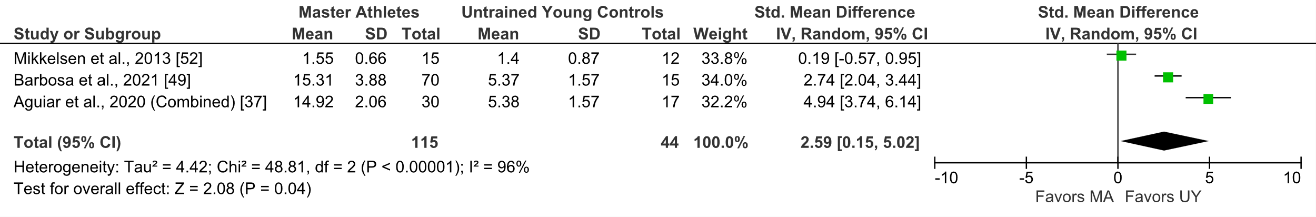


**Figure S4**. Subgroup analysis of TNF-α between master athletes and untrained young adults excluding female subjects. MA, master athletes; UY, untrained young controls


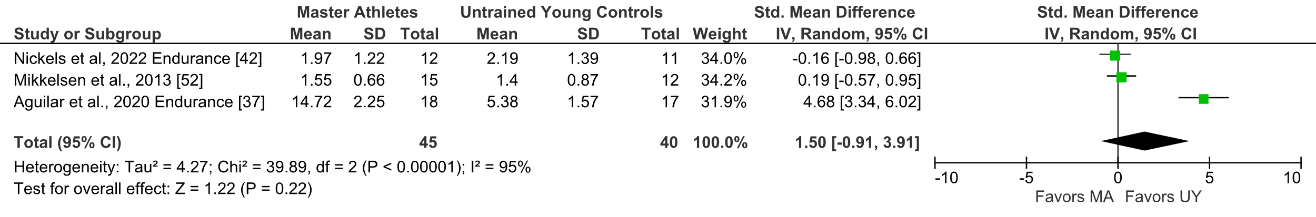


**Figure S5**. Subgroup analysis of TNF-α between master athletes and untrained young adults excluding non-endurance exercise athletes. MA, master athletes; UY, untrained young controls

**
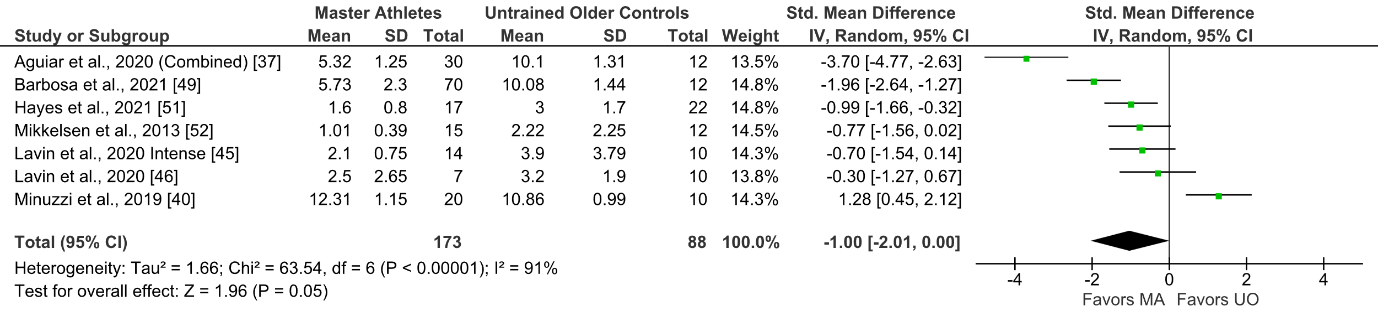
**

**Figure S6.** Subgroup analysis of IL-6 between master athletes and untrained middle-aged and older adults excluding moderate intensity athletes. MA, master athletes; UO, untrained middle-aged and older controls


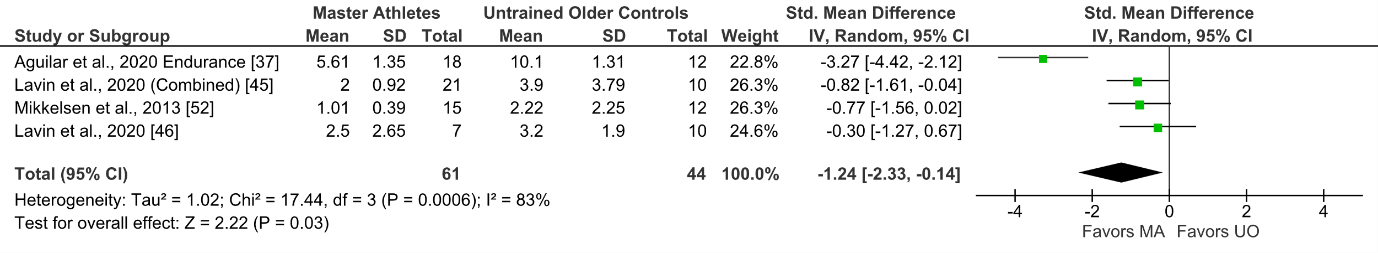


**Figure S7**. Subgroup analysis of IL-6 between master athletes and untrained middle-aged and older adults excluding non-endurance exercise athletes. MA, master athletes; UO, untrained middle-aged and older controls


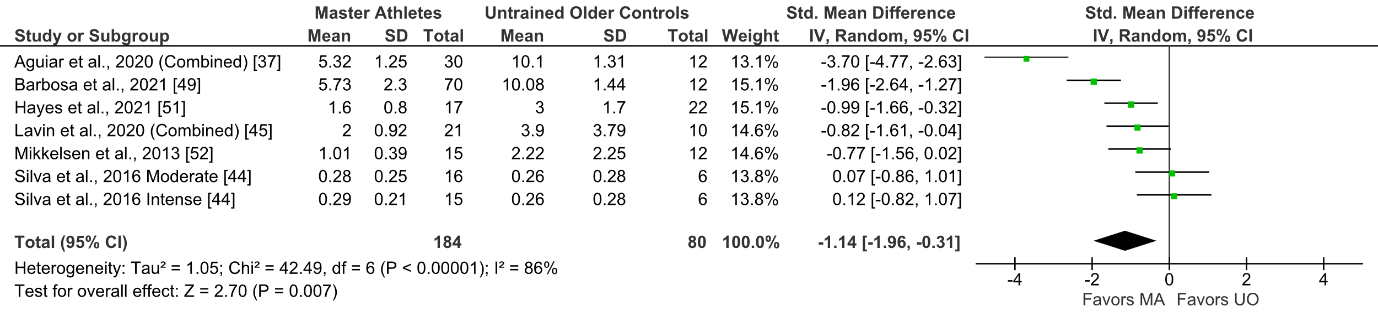


**Figure S8**. Subgroup analysis of IL-6 between master athletes and untrained middle-aged and older adults excluding female subjects. MA, master athletes; UO, untrained middle-aged and older controls


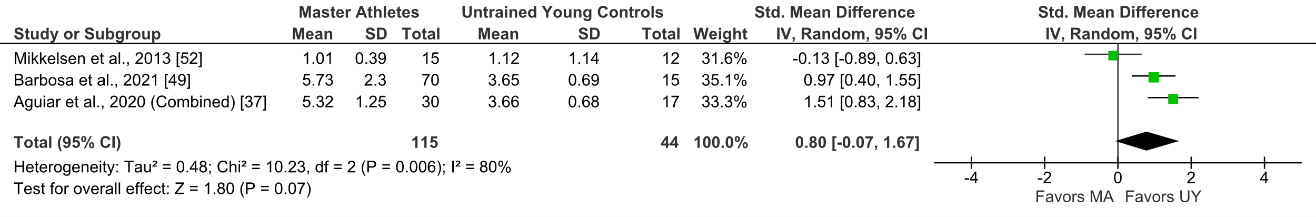


**Figure S9**. Subgroup analysis of IL-6 between master athletes and untrained young adults excluding female subjects. MA, master athletes; UY, untrained young controls


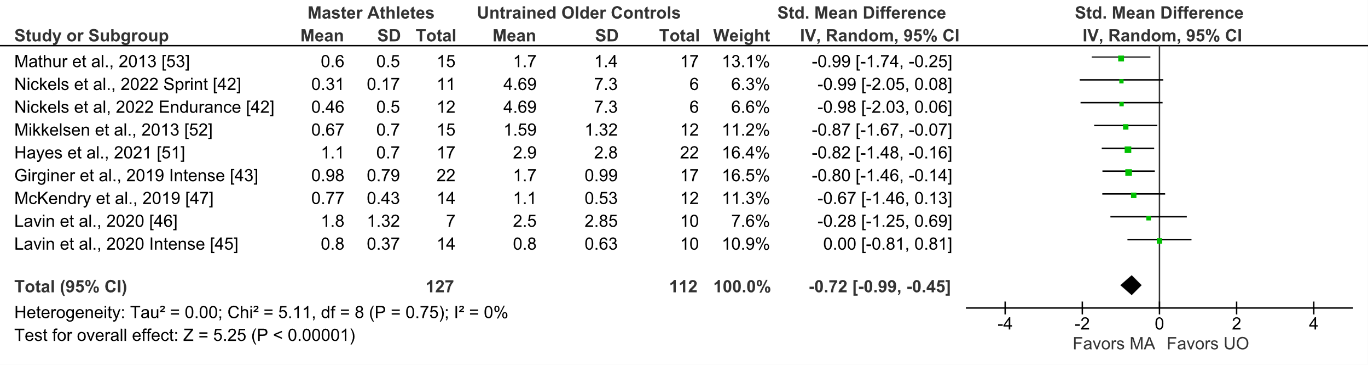


**Figure S10**. Subgroup analysis of CRP between master athletes and untrained middle-aged and older adults excluding moderate intensity athletes. MA, master athletes; UO, untrained middle-aged and older controls


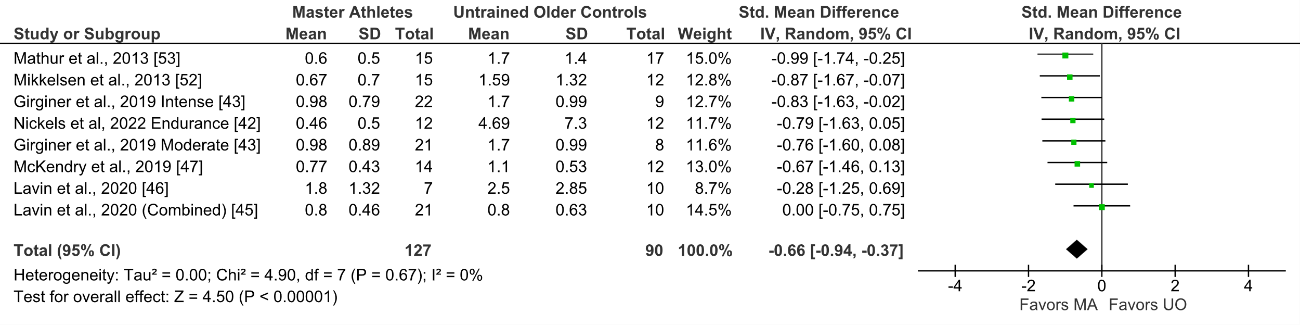


**Figure S11**. Subgroup analysis of CRP between master athletes and untrained middle-aged and older adults excluding non-endurance exercise athletes. MA, master athletes; UO, untrained middle-aged and older controls


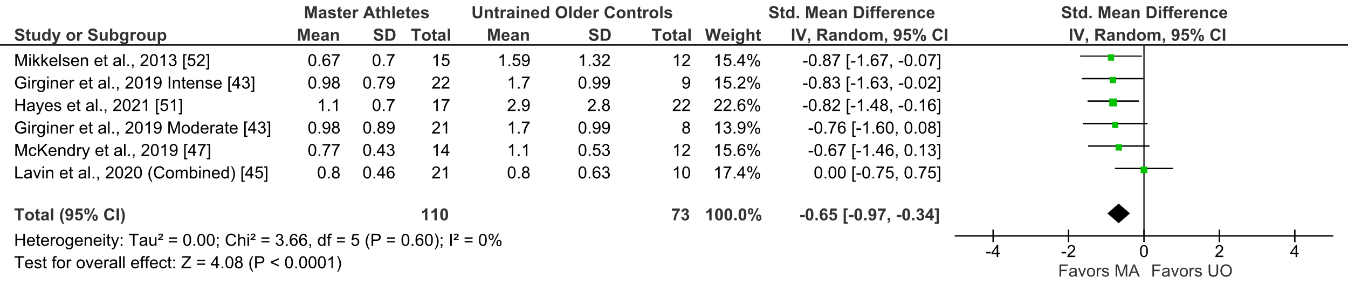


**Figure S12**. Subgroup analysis of CRP between master athletes and untrained middle-aged and older adults excluding female subjects. MA, master athletes; UO, untrained middle-aged and older controls


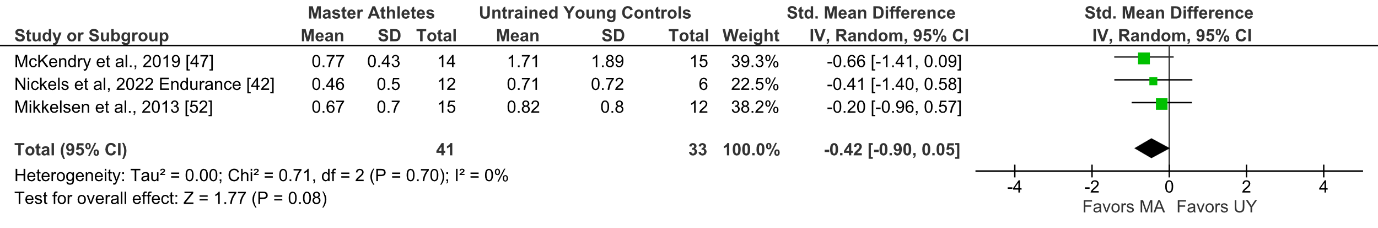


**Figure S13**. Subgroup analysis of CRP between master athletes and untrained young adults excluding non-endurance exercise athletes. MA, master athletes; UY, untrained young controls


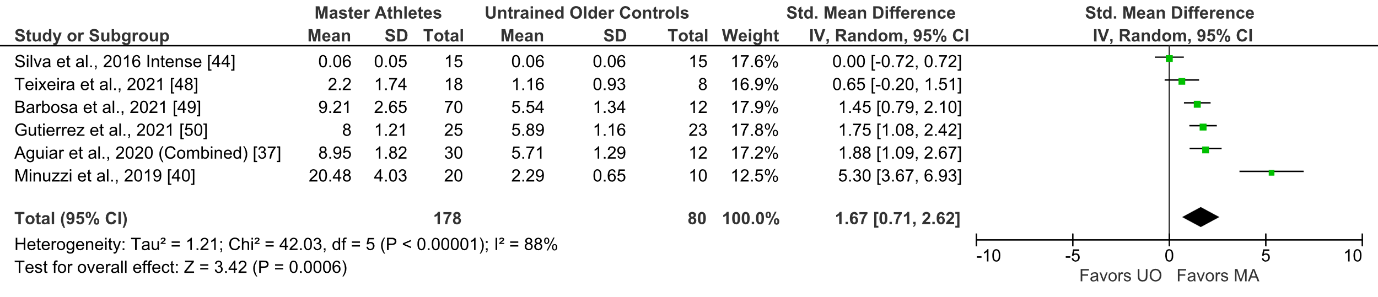


**Figure S14.** Subgroup analysis of IL-10 between master athletes and untrained middle-aged and older adults excluding moderate intensity athletes. MA, master athletes; UO, untrained middle-aged and older controls


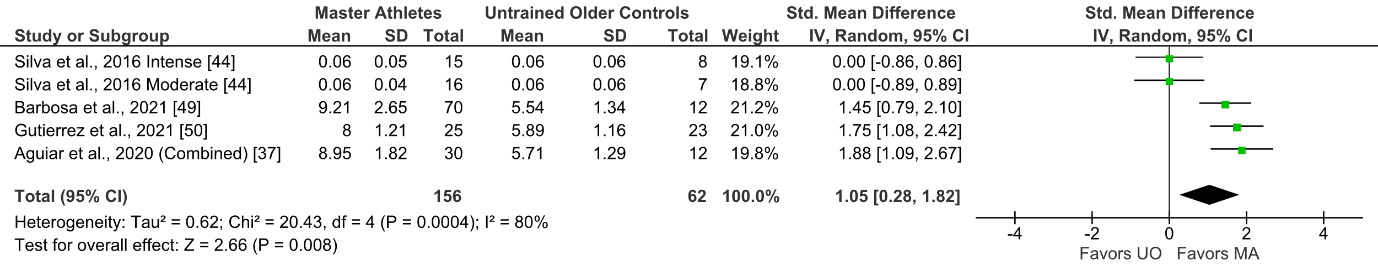


**Figure S15**. Subgroup analysis of IL-10 between master athletes and untrained middle-aged and older adults excluding female subjects. MA, master athletes; UO, untrained middle-aged and older controls


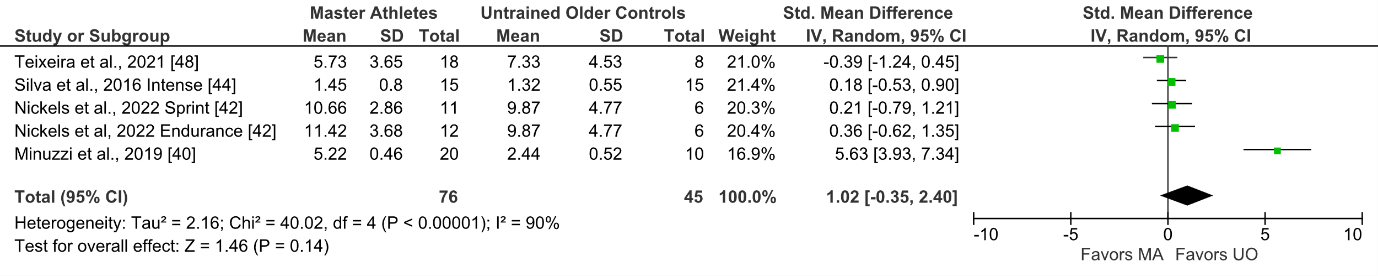


**Figure S16**. Subgroup analysis of IL-8 between master athletes and untrained middle-aged and older adults excluding moderate intensity athletes. MA, master athletes; UO, untrained middle-aged and older controls
